# *Drosophila* as a Model Organism to Study Basic Mechanisms of Longevity

**DOI:** 10.3390/ijms231911244

**Published:** 2022-09-24

**Authors:** Anna A. Ogienko, Evgeniya S. Omelina, Oleg V. Bylino, Mikhail A. Batin, Pavel G. Georgiev, Alexey V. Pindyurin

**Affiliations:** 1 Department of Regulation of Genetic Processes, Institute of Molecular and Cellular Biology SB RAS, 630090 Novosibirsk, Russia; 2 Laboratory of Biotechnology, Novosibirsk State Agrarian University, 630039 Novosibirsk, Russia; 3 Laboratory of Gene Expression Regulation in Development, Institute of Gene Biology RAS, 119334 Moscow, Russia; 4 Open Longevity, 15260 Ventura Blvd., Sherman Oaks, Los Angeles, CA 91403, USA

**Keywords:** longevity, aging, signaling pathway, IIS, TOR, NF-κB, JNK, gene expression regulation, *Drosophila*

## Abstract

The spatio-temporal regulation of gene expression determines the fate and function of various cells and tissues and, as a consequence, the correct development and functioning of complex organisms. Certain mechanisms of gene activity regulation provide adequate cell responses to changes in environmental factors. Aside from gene expression disorders that lead to various pathologies, alterations of expression of particular genes were shown to significantly decrease or increase the lifespan in a wide range of organisms from yeast to human. *Drosophila* fruit fly is an ideal model system to explore mechanisms of longevity and aging due to low cost, easy handling and maintenance, large number of progeny per adult, short life cycle and lifespan, relatively low number of paralogous genes, high evolutionary conservation of epigenetic mechanisms and signalling pathways, and availability of a wide range of tools to modulate gene expression in vivo. Here, we focus on the organization of the evolutionarily conserved signaling pathways whose components significantly influence the aging process and on the interconnections of these pathways with gene expression regulation.

## 1. Introduction

Aging is a fundamental process that is characteristic to some extent for all multicellular organisms and is most studied and understood in the evolutionary line of animals. In mammals, it is characterized by the functional loss of cells, tissues, and organs and is accompanied by the increasing susceptibility to many diseases, including cancer, neurodegeneration, cardiovascular diseases, and metabolic disorders of an organism [1]. One of the most striking manifestations of the aging of process, both in humans and *Drosophila*, is that gradual deregulation and a violation in the functioning of signal transduction pathways can lead to diseases and death of organisms. Changes in the functioning of signaling pathways are known to result in reduction or lengthening of lifespan. For example, genetic manipulations with the insulin/insulin-like growth factor 1 (IGF-1) signaling (IIS) pathway and target of rapamycin (TOR) signaling pathway that receive signals from growth factors and nutrients (nutrient sensing), with the c-Jun NH_2_-terminal kinase (JNK) pathway (nutrient sensing and stress resistance), as well as with nuclear factor kappa B (NF-κB) pathway (inflammation and immune response) are associated with an increase in lifespan both in *Drosophila* and mammals [2,3,4]. Thus, signaling pathways can be efficient targets for interventions aimed at changing the lifespan of organisms.

*Drosophila* is one of the most studied model organisms, and in this review we describe in detail the organization of IIS, TOR, NF-κB, and JNK signaling pathways and lifespan control in *Drosophila*. We also draw parallels between *Drosophila* and humans. The analysis carried out in the review reveals general patterns in the functioning of four signaling pathways and allows us to draw conclusions about the conservation of lifespan control mechanisms between *Drosophila* and mammals. We also provide an overview of the mechanisms of epigenetic control of gene expression associated with the activity of these signaling pathways, where known.

## 2. Advantages of *Drosophila* as a Highly Informative Model Organism for Aging Studies

### 2.1. Advantages and Similarities of Drosophila and Mammals

Investigations using mammalian models (e.g., mice) require a large number of animals to be housed, maintained and subjected to invasive testing and therefore raise both economic and ethical concerns. Furthermore, studies on the aging of mammals are limited by their long lifespan. That is why non-vertebrate model organisms (e.g., yeast, worms, fruit flies) are popular among researchers in the aging field. Since the beginning of the last century, when the first gene in the history of genetics was discovered in *Drosophila*, fruit fly has been used as a model organism to identify conservative basic biological processes [5]. The first studies in the field of aging on *Drosophila* were carried out in the second decade of the last century, and since then this model animal has been extensively used for aging research [6,7]. High level of conservation of signaling transduction pathways between *Drosophila* and humans made *Drosophila* an indispensable model organism for identifying the pathways responsible for lifespan extension, as well as for understanding the complexity of the aging processes in eukaryotic organisms. Lastly, *Drosophila* and humans have similar classes of transcription factors [8] and, thus, share the mechanisms of gene expression regulation.

### 2.2. Features of the Biology of Drosophila as an Object for the Study of Aging

*Drosophila* has homologs to ~75% of human disease genes [9] and ~60% of all fly genes are conserved in humans [10,11]. Unlike mammals, handling and maintenance of fruit flies is easy and inexpensive over multiple generations, and experiments on flies are ethically acceptable. Flies are characterized by high fecundity (about 200 offspring per female), which allows for analysis of any feature of interest over hundreds of individuals to avoid statistical anomalies. *Drosophila* practically possesses one significant advantage over mammalian models in the aging field, that is short life cycle and lifespan of ~60–70 days for laboratory isogenic wild-type strains and ~120–130 days for non-isogenic flies from natural populations [12]. *Drosophila* overwinters in nature in the adult stage in vegetable warehouses, etc., wherever the temperature reaches 16 °C. At this temperature, the duration of lifespan of adult flies is significantly lengthened [13].

Health and longevity of fruit flies are affected by various factors such as diet, temperature, genetic and environmental interventions. Sexual differentiation has also significant effects on fly longevity. For instance, it was shown that male and female flies respond differently to dietary restriction (DR) [14]. Greater increase in lifespan was observed in females than in males in response to DR. Similarly, mating status also affects longevity of flies. Virgin females have the longest lifespan and mated females have the shortest lifespan while males are less affected [15]. Among possible mechanisms contributing to sex-specific lifespans may be genetic differences between the sexes, hormonal differences, and behavior [16]. Genetic background may also contribute to differences in lifespan between compared fly lines [17,18]. Therefore, studies of interventions aimed at understanding or targeting aging should be carried out using the closest genetic background between the compared fly lines as possible. Ideally, for lifespan tests, all fly lines should have the same genetic background. For that, mutations and transgenes should be backcrossed at a minimum of 10 generations to one or (preferably) two well-characterized, commonly available wild-type inbred strains (reviewed in [19]). Moreover, the compared fly lines should be free of the cytoplasmic endosymbiont, *Wolbachia*, since this infection can influence lifespan [20].

### 2.3. Statistical Analysis in Drosophila Aging Studies

Despite the fact that lifespan assays involve the simple counting of dead flies over time, it can be complicated by inconsistencies in approaches to estimating survival curves and other statistics. For instance, standard metrics in lifespan assay are the assessment of mean, median, or maximum lifespans within a *Drosophila* population. This is problematic, since variations in metrics between laboratories are quite common, leading to difficulties in comparison of lifespan effects. That is why comparison between survival curves of experimental and control flies and calculation of the significance level of differences between them (P) are performed using Kaplan–Meier method, log-rank test or Kolmogorov–Smirnov test [21].

### 2.4. Drosophila as a Model in the Study of Epigenetic Processes

Since the molecular mechanisms of gene regulation are rather evolutionarily conserved, *Drosophila* may be also a useful for studying the epigenetic mechanisms of gene expression control involved in development of aging and longevity (histone methylation and acetylation, microRNA expression, etc.). Epigenetic alterations in *Drosophila* are known to be controlled by the diet [22], temperature, illumination, and different forms of stress (heat shock, starvation). Therefore, *Drosophila* can be used as a model to study how exactly environmental stimuli affect epigenetic alterations. Understanding of how gradual deregulation of regulatory systems and imbalance of epigenetic mechanisms of gene regulation result in aging progression will contribute to a faster concretization of approaches aimed at combating aging. Thus, *Drosophila* turned out to be a convenient object for studying epigenetic mechanisms of longevity and aging.

### 2.5. Limitations in Using Drosophila as a Model in Aging Research

*Drosophila*, of cause, is one of the best analyzed/understood multicellular organism, more complex in comparison with *C*. *elegans* and yeast and is an ideal model system due to ease of genetic and environmental manipulations, completely sequenced genome, relatively low number of paralogous genes, the opportunity to investigate influence of the parameters such as gender ratio, resistance to pathogens, chemicals and environmental stress factors on longevity. In addition, *Drosophila* does not have programmed aging, as, for example, *Arabidopsis*, in which the death of an adult organism is associated with the maturation of seeds [23]. In *Drosophila*, aging apparently occurs as in humans: survival curves and mortality curves in these species look the same; moreover, the peak of reproduction in both species is shifted to early life periods [24]. However, several restrictions of using *Drosophila* exist as well. First, *Drosophila* goes through different developmental stages (embryo, larva, pupa, and adult) which mammals do not have [25]. Second, *Drosophila* differs substantially from mammals at the organismal level, brain anatomy, cardiovascular system, and respiration systems. Third, flies have a less complex and adaptive immune system than mammals and do not maintain their body temperature. This can lead to the fact that the effects of drugs/inhibitors/geroprotectors can vary greatly between *Drosophila* and mammals (the conversion of protoxins to toxins in the liver, as an example).

From a molecular point of view, despite the general good conservation of basic signaling pathways and cellular processes between mammals and *Drosophila*, low redundancy/reduced number of paralogous genes in *Drosophila* in comparison to mammals does not allow reproducing on this organism the regulatory complexity that is inherent in mammals, in which paralogization of regulatory genes is one of the main mechanisms for acquiring complexity [26,27,28]. In addition, poor conservation of some proteins/protein functions may be a problem as well.

Nevertheless, the comprehensibility (recognizability) of *Drosophila* as a model organism, as well as the rapid rate of generation turnover in this species, coupled with the conservation of the main regulatory and signaling pathways, makes *Drosophila* indispensable in aging studies. Below, we look in detail at the main signaling pathways in *Drosophila* known to be important for controlling aging.

## 3. Signaling Pathways Regulating *Drosophila* Lifespan

Below we briefly describe, from the *Drosophila* point of view and concentrating on the extracellular signals–gene expression regulation axis, evolutionary conserved signaling pathways, which dysfunctions result in altered fly lifespan. Thereby, direct effects on cellular protein synthesis, e.g., through modulation of activity of translation initiation factors that are not mediated by changes in expression level of genes encoding them, are not covered. More in-depth information on the signaling pathways, including their peculiarities in different species from yeast to humans as well as descriptions of their branches not directly related to gene expression regulation, is available in other reviews (see references below).

### 3.1. Insulin/IGF-1 Signaling (IIS) Pathway

**Figure 1:**
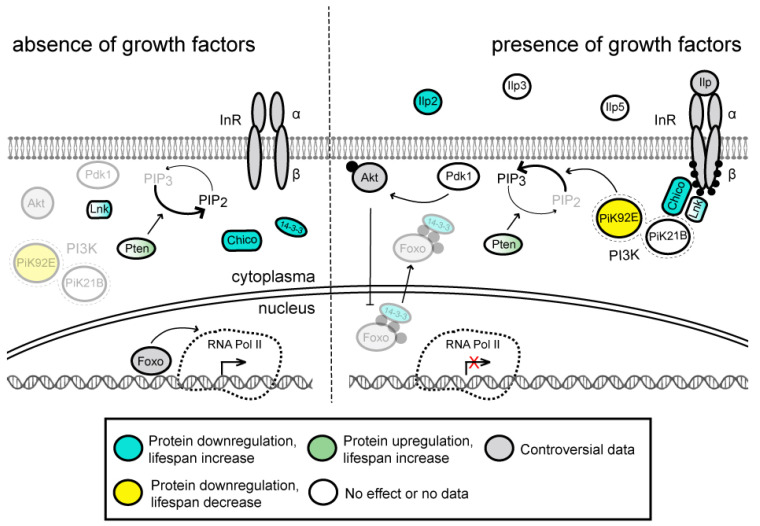
*Drosophila* insulin/IGF-1 signaling (IIS) pathway, a simplified schematic representation. The inactive and active states of the pathway are shown on the left and right, respectively. The binding of the insulin-like peptides (Ilp1-7) to insulin-like receptor (InR) initiates a phosphorylation cascade that results in the regulation of metabolism. Chico and Lnk, insulin receptor substrates; PI3K, phosphatidylinositol 3-kinase consisting of two subunits, catalytic Pi3K92E (Dp110) and regulatory Pi3K21B (Dp60); PIP_2_, phosphatidylinositol 4,5-bisphosphate; PIP_3_, phosphatidylinositol 3,4,5-trisphosphate; Pten, phosphatase and tensin homolog; Pdk1, 3-phosphoinositide dependent protein kinase-1; Akt, protein kinase B (PKB); Foxo, transcription factor Forkhead box O; 14-3-3, chaperone proteins. Arrows and bar-headed lines indicate activation and inhibition, respectively. Phosphate groups are depicted as solid black dots. Protein complexes are encircled by dashed lines. Inactive components of the pathway are shown in semi-transparent mode. Effects of mutations, depletion or overexpression of protein molecules on adult fly lifespan are color-coded according to the legend at the bottom; different simultaneous effects are shown as color gradients (for the references, see Supplementary Table S1).

The insulin/insulin-like growth factor 1 (IGF-1) signaling (IIS) pathway is involved in regulation of various physiological processes, including growth, development, metabolism, stress responses and reproduction (reviewed in [29,30,31,32]) (Figure 1). In total, hundreds of proteins are involved in the *Drosophila* insulin network [33]. The pathway is activated when one of the seven *Drosophila* insulin-like peptides (Ilp1–Ilp7, also known as DILP1–DILP7; information about human ortholog(s) of the fly proteins is provided in Supplementary Tables S1–S4) [34,35] binds to the insulin receptor (InR) [36,37,38] on the surface of the target cells.

This interaction stimulates tyrosine-protein kinase activity of InR [34,37,39] and results in a conformational change of the latter followed by its autophosphorylation and subsequent autoactivation [40]. Activated InR conveys signals further to Pi3K21B (Dp60) by its direct phosphorylation and through phosphorylation of the insulin receptor substrates Chico and Lnk that interact with Pi3K21B [41,42,43]. Regulatory Pi3K21B subunit together with catalytic Pi3K92E (Dp110) subunit form PI3K kinase that is responsible for conversion of phosphatidylinositol (3,4)-bisphosphate (PIP_2_) into phosphatidylinositol (3,4,5)-trisphosphate (PIP_3_) [44]. PIP_3_ serves as second messenger, activating protein kinase Pdk1, which in its turn phosphorylates protein kinase Akt (also known as PKB) at Thr342 and thus activates it [45,46,47,48]. Activated Akt phosphorylates transcription factor Foxo, which is responsible for upregulation of genes involved in longevity and stress resistance [49,50] (for details see below). As a result, phosphorylated Foxo becomes inactivated by nuclear exclusion with the help of 14-3-3ζ and 14-3-3ε chaperone proteins [51,52,53]. Regulation of Foxo activity by insulin/IGF pathway is evolutionarily conserved in humans [54,55], in which 14-3-3 protein promotes sequestering and inactivation of human FOXO3a after its phosphorylation by kinases such as Akt [56,57]. The IIS pathway is antagonized by phosphatase activities of Pten and PP2A, which dephosphorylate PIP_3_ and Akt, respectively [58,59,60,61,62,63] (Figure 1).

**Figure 2:**
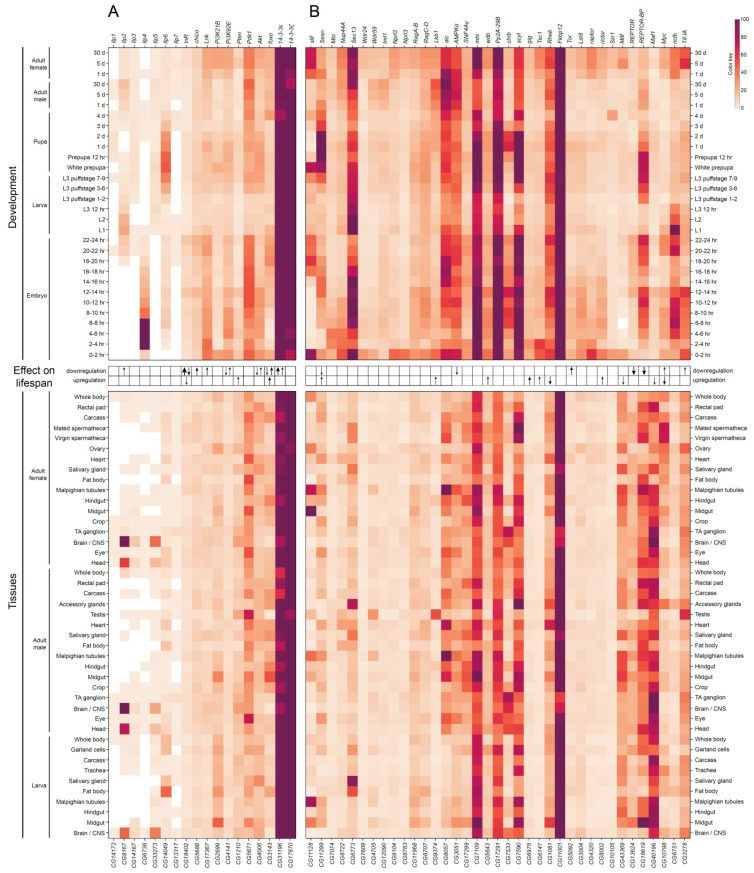
Heat map representation of the expression patterns of genes encoding components of *Drosophila* insulin/IGF-1 signaling (IIS) (**A**) and target of rapamycin (TOR) signaling (**B**) pathways during developmental stages (at the top) and across different tissues/organs (at the bottom). Gene expression data were taken from modENCODE and FlyAtlas 2 databases [64,65]. White and deep violet colors represent low and high gene expression levels, respectively. In addition, a summary of effects on fly lifespan caused by gene mutations, ubiquitous or tissue-specific knockdown or overexpression is shown between the gene expression top and bottom heat maps. Up and down arrows indicate increased and decreased adult fly lifespan, respectively; the size of arrows reflects the magnitude of the effect (for the references, see Supplementary Tables S1 and S2).

Some components of the IIS pathway are expressed at undetectable/very low to moderate levels with minimal variability across developmental stages and different tissues, for example, Ilp1, Ilp7, InR and Chico (Figure 2A and Supplementary Figure S1A). Other components generally expressed at very low to moderate levels are characterized by relatively high abundance at specific developmental stages and tissues. Among those are Ilp4, Ilp2 and Pi3K21B, which are actively expressed in early embryonic stages, larval/adult central nervous system (CNS) neuronal cells, and larval/adult digestive system, respectively. The 14-3-3 proteins are the only ones that are expressed at relatively stable extremely high levels, most probably due to their involvement in many other cellular processes apart from the IIS pathway. The IIS pathway can be functionally combined with the TOR pathway, so we summarize the conclusions from the heat map analysis drawn in Figure 2B and Supplementary Figure S1B in the TOR section (see below).

Downregulation of several pathway components that negatively regulate Foxo activity was shown to extend *Drosophila* lifespan (Supplementary Table S1). First, it is necessary to mention that thorough analysis of viable *Ilp1*-*Ilp7* null mutants uncovered that most of the Ilps can act redundantly. Individual knockout of Ilp2, which has the highest homology to human insulin (IGF1, IGF2), results in a median adult lifespan extension of 9% and 13% for males and females, respectively [35]. Similarly, partial genetic ablation of the neurosecretory Ilp-producing cells that reside in the brain and are the primary producers of Ilp2, Ilp3 and Ilp5 in the adult body resulted in median lifespan increase by 10.5% in males and 18.5% and 33.5% in virgin and mated females, respectively [66]. Second, some *InR*, *chico* and *14-3-3ε* mutations also extend lifespan [38,39,52,67,68]. Particularly, transheterozygous *InR^05545^*/*InR^E19^* females demonstrated adult lifespan extension of 85%, while other homo- and transheterozygous *InR* mutants were found to be strongly or moderately short-lived [39]. Heterozygotes *chico^1^* males and females showed increase of median adult lifespan of 13% and 36%, respectively. At the same time, the effect of homozygous *chico^1^* mutation on the adult lifespan is completely different in males and females; the former are slightly short-lived, whereas the lifespan of the latter is extended by 48% [68].

Similarly, the *Drosophila* lifespan can be extended by upregulation of antagonistic IIS pathway regulators. Thus, Pten or Foxo overexpression in adult head fat body or adult male muscles increased the median lifespan of flies [69,70]. In addition, the extended lifespan was observed upon Foxo overexpression in female, but not male, adult fat body [71]. Interestingly, overexpression of Foxo or Pten in other tissues, e.g., in adult CNS or heart, had no positive effect on the lifespan [69,72]. Thus, the role of Foxo may be different depending on the tissue; in some cases, as for example in the CNS or dorsal vessel higher levels of Foxo have no effect on the longevity, but in the fat body or muscles it causes a life extension. Probably, the positive effect of Foxo on the lifespan is associated with maintenance of muscle tissue or with activation of lipases and mobilization of fat in fat body [73,74].

Several homozygous loss-of-function mutations or transheterozygous combinations of loss-of-function and hypomorphic mutations of the IIS pathway genes, e.g., *InR^05545^*/*InR^E19^*, *chico^1^*/*chico^1^* and *Akt^1^*/*Akt^1^*, result in delayed development and a dwarf phenotype due to decreased cell number and cell size [34,42,75,76,77,78], which is even reflected in the name of the *chico* gene that means “small boy” in Spanish. In addition, the extension of female lifespan by modulating the expression level of the IIS pathway genes is often associated with reduced or delayed fecundity [38,42,66,68,71]. However, either dwarfism or reduced fecundity might not be necessarily coupled to the lifespan extension [68,69,79,80]. Moreover, in addition to Foxo, some other factors are very likely to be involved in the IIS pathway-mediated small body size and fecundity defects [78].

Thus, it can be concluded that the IIS pathway performs a “pro-nutritional role”. It activates/suppresses protein synthesis and cell proliferation through the TOR signaling pathway (see below) in response to the presence of food/fasting conditions, respectively, and, at the same time, it suppresses cell proliferation through Foxo, mobilizing fat reserve in adipose tissues via Foxo activity upon fasting. The effects of lengthening lifespan upon suppression of the IIS signaling pathway may be associated with the redistribution of nutrients under fasting conditions, as well as slowing down cell divisions with a decrease in protein synthesis.

### 3.2. Target of Rapamycin (TOR) Signaling Pathway

**Figure 3:**
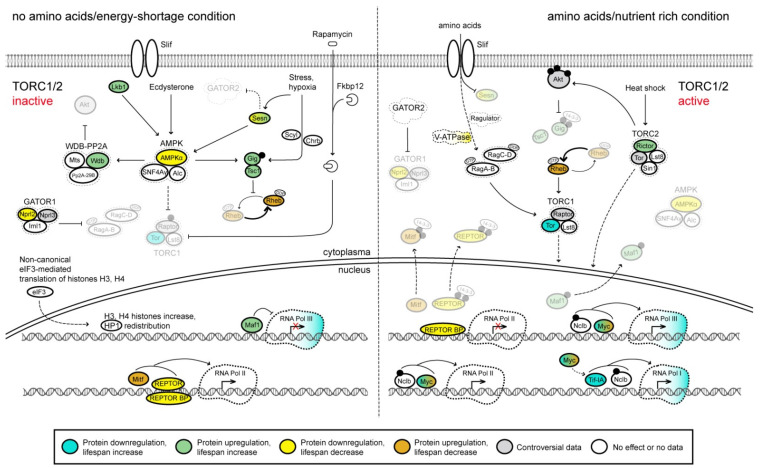
*Drosophila* target of rapamycin (TOR) signaling pathway, a simplified schematic representation. The inactive and active states of the pathway are shown on the left and right, respectively. The anabolic kinase TOR functions in two distinct complexes, TORC1 and TORC2, and regulates cap-dependent translation as well as chromatin architecture and gene regulation. Through a number of intermediate steps, nutrients, particularly amino acids, and growth factors, such as insulin-like peptides, activate TORC1 (for additional details, see Figure 1). Akt, protein kinase B (PKB); Alc, Alicorn; AMPK, AMP-activated protein kinase; Chrb, Charybde; Fkbp12, FK506-binding protein 12kD; GATOR, GAP activity towards the Rags; Gig, Gigas; HP1, heterochromatin protein 1; Iml1, Increased minichromosome loss 1; Lkb1, Lkb1 kinase; Mts, Microtubule star; Nclb, No child left behind; Nprl2, Nitrogen permease regulator-like 2; Nprl3, Nitrogen permease regulator-like 3; Pp2A-29B, Protein phosphatase 2A at 29B; RagA-B, Ras-related GTP binding A/B; RagC-D, Ras-related GTP binding C/D; REPTOR, Repressed by TOR; REPTOR-BP, REPTOR-binding partner; Rheb, Ras homolog enriched in brain; Rictor, Rapamycin-insensitive companion of Tor; Scyl, Scylla; Sesn, a stress-inducible protein Sestrin; Sin1, SAPK-interacting protein 1; Slif, Slimfast; SNF4Aγ, SNF4/AMP-activated protein kinase gamma subunit; Tor, Target of rapamycin; V-ATPase, vacuolar-type ATPase; Wdb, Widerborst; 14-3-3, chaperone proteins. For the other proteins shown in the scheme, the symbols and the full names are identical. Arrows and bar-headed lines indicate activation and inhibition, respectively. Dotted arrows indicate that the molecular mechanisms are not fully understood. Phosphate groups are depicted as solid black dots. Protein complexes are encircled by dashed lines. Inactive components of the pathway are shown in semi-transparent mode. It should be noted that the indicated transcription factors might not always be required together for the activity of the target genes. Effects of mutations, depletion or overexpression of protein molecules on adult fly lifespan are color-coded according to the legend at the bottom; different simultaneous effects are shown as color gradients (for the references, see Supplementary Table S2).

Being interconnected with the IIS pathway, the target of rapamycin (TOR) signaling pathway coordinates cell growth responses to the availability of nutrients and energy (reviewed in [81,82,83,84,85,86]) (Figure 3). Rapamycin is a natural compound with antifungal activity originally isolated from *Streptomyces hygroscopicus* bacteria [87]. It specifically inhibits Tor activity within TORC1, but not TORC2, complex in a wide range of organisms including *Drosophila* [88,89]. This property of rapamycin is reflected in the names of the Tor protein and the entire signaling pathway. The central component of the TOR pathway is the Ser/Thr kinase Tor, which functions in two distinct multiprotein complexes: Tor complex 1 (TORC1) and Tor complex 2 (TORC2) [90,91]. TORC1 consists of Tor, Lst8 and Raptor, while TORC2 consists of Tor, Lst8, Rictor and Sin1 [92,93,94]. The TORC1 activity is regulated by different upstream signals including, but not limited to, amino acids [95,96], growth factors via the Akt kinase (reviewed in [97]), signaling through AMP kinase (AMPK) pathway [98,99] and stress response through the regulation of the tuberous sclerosis complex (TSC) [100]. Much less is known about TORC2 regulation; in particular, this complex is activated upon heat stress [101,102] (Figure 3). It is important to mention that besides phosphorylating and thus regulating the activity of several cytoplasmic proteins, both Tor complexes were found to influence nuclear transcriptional programs, although much more evidence is available for TORC1 [103,104,105,106,107,108,109,110].

Briefly, when cells have sufficient amino acids, TORC1 is recruited from the cytoplasm to the lysosomal surface, where it is subsequently activated [111,112]. Each of these processes is described in more detail below. Amino acids imported into the cell by specialized transporters such as Slimfast (Slif) [113,114,115] are sensed by a number of proteins. One of them is Sestrin (Sesn) that in the presence of leucine and the absence of stresses dissociates from the multisubunit GATOR2 complex and, thus, activating it [116,117] (reviewed in [118,119]). GATOR2 is an inhibitor of GATOR1 complex, which is a GTPase-activating protein (GAP) toward the Ras-related GTP binding A/B (RagA-B) protein [116]. Inactivation of GATOR1 by GATOR2 allows RagA-B in the form of a heterodimer with RagC-D protein to target TORC1 to lysosomes [120,121]. At the lysosomal membrane, TORC1 can be subsequently activated by the GTPase Rheb [112,122] (Figure 3). TSC, which consists of the Tsc1 and Gigas (Gig) proteins and senses several different inputs including the presence of growth factors, energy and other stresses, is another important regulator of TORC1 activity (reviewed in [123]). In the absence of growth factors, TSC negatively regulates TOR signaling [124,125,126]. Upon activation of the IIS pathway, Akt phosphorylates both TSC components, although only Akt-dependent phosphorylation of Gigas at Ser924 and Thr1518 is conserved in mammals [127,128]. Based on the knowledge on mammalian counterparts, this likely results in acute sequestration of TSC from the lysosomal membrane to the cytoplasm by 14-3-3 chaperone proteins [129] (reviewed in [123]). Since Gigas is a GAP specific for Rheb, depletion of TSC at the lysosomal surface results in activation of Rheb, which is a key upstream activator of TORC1 [101,111,112,130,131]. The TORC1 activity is also indirectly regulated by another Tor complex, TORC2, which phosphorylates, among other targets, Akt at Ser505, thus increasing its kinase activity [101,132,133,134,135]. However, it is necessary to note that the functional significance of Akt-dependent phosphorylation of TSC in *Drosophila* is still not completely clear, as simultaneous mutations of the appropriate phosphorylation sites in the Tsc1 and Gigas proteins were reported to not affect the viability and development of flies [128,136], indicating the possible existence of redundant regulatory mechanism.

On the contrary, the TORC1 activity is inhibited by the AMPK complex, rapamycin and stress conditions like hypoxia and starvation (Figure 3). AMPK consists of the three components, AMPKα, Alicorn (Alc) and SNF4/AMP-activated protein kinase gamma subunit (SNF4Aγ) and is the main sensor of cellular energy status [137,138,139]. More specifically, this complex is activated by low energy levels (high AMP/ATP and ADP/ATP ratios) and then it phosphorylates, among other targets, Gigas at Ser1338 resulting in its functional activation [99]. In human cells, AMPK also directly phosphorylates Raptor, a regulatory component of TORC1 that leads to downregulation of TORC1 activity [98]. A high degree of conservation of the AMPK target site in Raptor suggests that AMPK might directly inhibit TORC1 in *Drosophila* cells as well [98]. Thus, AMPK inhibits TORC1 indirectly via TSC and Rheb and also possibly directly. In addition, high levels of steroid hormone 20-hydroxyecdysone, which controls developmental transitions in *Drosophila*, results in AMPK-dependent activation of heterotrimeric PP2A complex and, subsequently in inhibition of Akt activity [140]. On the other hand, the AMPK activity was shown to be positively dependent on Sestrin and the Lkb1 kinase [141,142]. Although the molecular mechanism of rapamycin-dependent TORC1 inhibition in *Drosophila* cells has not been elucidated yet, it likely involves intracellular FK506-binding proteins, such as Fkbp12, similarly to yeast and mammalian cells [143,144,145]. At present, rapamycin is most well-studied and most specific TORC1 inhibitor; its addition to fly food extends *Drosophila* lifespan [89,146,147]. The Scylla (Scyl) and Charybde (Chrb) proteins, both of which are upregulated under hypoxia or starvation, contribute to the activation of TSC, thus inhibiting TORC1 [148].

TORC1 regulates cell growth and adult fly lifespan in part through phosphorylation of cytoplasmic proteins that control protein synthesis and autophagy [89,149]. Consistently, changes in the expression levels of, for example, the eIF4E-binding protein (Thor), an inhibitor of cap-dependent protein synthesis, and ribosomal protein S6 kinase (S6k) that both are TORC1 targets, were shown to affect cells growth and *Drosophila* lifespan [150,151,152,153].

It was convincingly shown that activity of some transcription factors is regulated by TORC1, although the detailed molecular mechanisms regulating their activity are not always known (Figure 3). Among these transcription factors are Maf1, REPTOR and Mitf, which are active only when TORC1 is inactive. Maf1 is a repressor of RNA polymerase (RNA Pol) III activity. REPTOR together with its binding partner REPTOR-BP is required for expression of the vast majority of protein-coding genes, whose activity is induced by rapamycin treatment (promotes animal survival upon nutrient restriction) [107]. Mitf is a master regulator of transcription of genes encoding components of the vacuolar (H^+^)-ATPase (V-ATPase) holoenzyme that constitutes a part of the TOR signaling pathway at the lysosome membrane (not described above; see Figure 3) [108]. Activation of TORC1 eventually leads to phosphorylation of REPTOR, and presumably of Mitf, and their subsequent export from the nucleus to the cytoplasm by 14-3-3 proteins [107,108]. According to data available for Maf1 orthologs in other species, the similar mechanism could also work for *Drosophila* Maf1 [109,154,155]. In the absence of TORC1 activity, Maf1, REPTOR and Mitf become dephosphorylated by PP2A phosphatases and return to the nucleus, where they activate transcription of their target genes [107,108,155]. Contrariwise, transcription factors Myc, Nclb and Tif-IA are positively regulated by TORC1. Myc and Nclb (also known as PWP1) promote transcription mediated by all nuclear RNA Pols; although in the case of Myc and RNA Pol I the effect seems to be indirect and mediated, in part, by Tif-IA [104,105,110,115,156,157,158,159,160]. At least in the case of Nclb, it was found that this transcription factor is phosphorylated in a TORC1-dependent manner [110]. Overall, upon TORC1 activation, the activity of genes primarily involved in ribosome biogenesis is significantly upregulated. Among these genes are ribosomal, transfer and small nucleolar RNAs transcribed by RNA Pols I and III, as well as genes encoding ribosomal proteins that are transcribed by RNA Pol II. At the same time, transcription of autophagy-related genes (e.g., those encoding lysosomal proteins) becomes repressed. Besides that, inactivation of TORC1 results in elevated expression levels of histones H3 and H4 through non-canonical translation mediated by eIF3 [161] (Figure 3). More specifically, treatment of *Drosophila* with rapamycin increases levels of the histone proteins as well as the number of nucleosomes, ultimately altering chromatin structure and activating autophagy genes at least in the intestine [161]. Finally, TORC2 controls the nuclear localization and thus activity of Myc [106] as well as negatively regulating Foxo activity through Akt phosphorylation [132,134] (Figure 1 and Figure 3).

Similarly to the genes encoding the components of the IIS pathway, tissue-specific and developmental expression profiles of the TOR signaling pathway genes are not uniform (Figure 2B and Supplementary Figure S1B). The majority of genes encoding GATOR1, GATOR2, TORC1 and TORC2 components are ubiquitously expressed at very low to moderate levels. At the same time, a number of genes of the pathway exhibit tissue-specific expression patterns. For example, the amino-acid transporter gene *slimfast* is specifically expressed in the digestive tract, Malpighian (renal) tubules, ovaries and testes, while the *Secretory 13* (*Sec13*) gene encoding a component of GATOR2 is predominantly active in salivary glands, fat body, accessory glands and spermathecae. The catalytic and scaffold subunits of the PP2A phosphatase complex, Mts and PP2A-29B, are both expressed at high levels during *Drosophila* development, probably due to the involvement of this complex in many different cellular processes. Lastly, transcription factors regulated by the TOR pathway demonstrate high variability in their tissue-specific and developmental expression profiles (Figure 2B and Supplementary Figure S1B).

Importantly, in all tissues and developmental stages, the same pattern of rather strongly expressed genes is observed. The results obtained for the transcriptome (Figure 2B and Supplementary Figure S1B) and proteome (Supplementary Figure S1B) are somewhat inconsistent, partly due to the lack of data for some proteins in the proteome, but by comparing them we can confidently say that at least both forms of chaperon 14-3-3 (promotes exclusion of phosphorylated Foxo from the nucleus), Sec13 (component of GATOR2, which activates the TORC1 signaling pathway through the inhibition of the GATOR1, thus, controlling the switch to cell proliferation and growth when food available and during female oocyte development), Mts, PP2A-29B, Wdb (catalytic, scaffold and regulatory subunit of PP2A phosphatase, respectively, which dephosphorylate Akt kinase, antagonizing the IIS pathway activity), and Fkbp12 (inhibitor of TOR) are ubiquitously expressed. Thus, it can be summarized that in all tissues and at all stages of development, a set of proteins is expressed that contributes to the activation of metabolism and protein synthesis upon food intake (14-3-3, GATOR2), and a set of proteins that contributes to the suppression/inactivation of metabolism and protein synthesis upon fasting (Mts, PP2A-29B, Wdb, Fkbp12). We concluded that the IIS and TOR pathways are always ready for both activation and repression in response to changing environmental conditions in terms of the availability of food resources. This may play a large adaptive role in the life of *Drosophila*, since the high energy expenditure associated with flight forces the flies to consume food frequently.

Inactivation of TORC1 caused by rapamycin treatment substantially increases the lifespan of *Drosophila* females and to a lesser extent of males [89]. Interestingly, the effect of rapamycin on the lifespan can be further enhanced by particular mutants of the IIS pathway (e.g., heterozygous *chico^1^* background) as well as by DR [89]. Also, it was found that rapamycin treatment results in improved gut health in aged flies, in part through autophagy induction [147] and reduction of precursor tRNA (pre-tRNA) levels, suggesting the involvement of RNA Pol III [109]. Noticeably, gut-specific overexpression of Maf1, a key repressor of RNA Pol III-mediated transcription, slightly extends female lifespan [109]. Furthermore, knockdown of individual subunits of RNA Pol III either in the entire gut or specifically in gut intestinal stem cells (ISCs) prevents functional decline of the gut in aged flies as well as extending lifespan of flies; the latter effect is again much stronger for females then for males [109]. In this connection, it is necessary to mention that the homeostasis of ISCs was shown to be critical for *Drosophila* longevity [162].

*Tor^2L7^*/*Tor^k17004^* transheterozygous mutant flies with reduced activity of both TORC complexes are characterized by 20% longer median lifespan as well as a smaller body size than their counterparts carrying *Tor^+^* rescue transgenic construct [163]. This situation is similar in mammals, where mutations in growth hormone or growth hormone receptor in mice cause a dwarf phenotype and extended lifespan [164,165]. Accordingly, genetic inactivation and overexpression of negative regulators of TORC1 result in shortened and extended adult fly lifespan, respectively (Supplementary Table S2). For example, ubiquitous RNAi-mediated knockdown of the *AMPKα* gene and null mutations of the *Sestrin* and *Nprl2* genes (the latter one encodes a subunit of the GATOR1 complex) were each shown to shorten the lifespan of *Drosophila* [117,166,167]. In addition, ubiquitous or/and tissue-specific overexpression of Sestrin, Lkb1, Tsc1 or Gig increases the lifespan [117,142,152].

Besides Maf1 described above, other transcription factors regulated by TORC1 also contribute to the fly lifespan. First, *REPTOR* and *REPTOR-BP* knockout animals of both sexes have drastically reduced lifespan [107]. Second, *Mitf* overexpression in the nervous system leads to ~10% decrease in the lifespan of females and flies were not viable when *Mitf* was overexpressed in all tissues [168]. Third, *Myc* overexpression and haploinsufficiency substantially shortens and extends the lifespan, respectively [169]. Finally, single mutations in the *Tif-IA* and *Polr1A* genes encoding the essential activator of RNA Pol I and the largest specific subunit of this polymerase, respectively, were shown to extend the median lifespan by 8%, at least in females [170].

Thus, inactivation of transcription factors that promote animal survival upon nutrient restriction (REPTOR and REPTOR-BP), as well as an increase in the synthesis of ribosomal RNA genes due to Myc, reduces lifespan. On the contrary, moderate lengthening of lifespan results from a decrease in the expression of transcription factors that promote the synthesis of ribosomal RNA (Tif-IA or Polr1A). The negative effects of *Mitf* overexpression (induces lysosomal vacuolar (H^+^)-ATPase genes, essentially autophagy genes) are more difficult to explain: on the one hand, autophagy activation in neurons can be harmful to flies, and on the other hand, additional expression of lysosomal vacuolar (H+)-ATPase proteins may increase the pool of Rag GTPases associated with them on the lysosomal membrane, and these could activate TORC1 more readily leading to a subsequent drop in lifespan.

### 3.3. NF-κB Signaling Pathway

**Figure 4:**
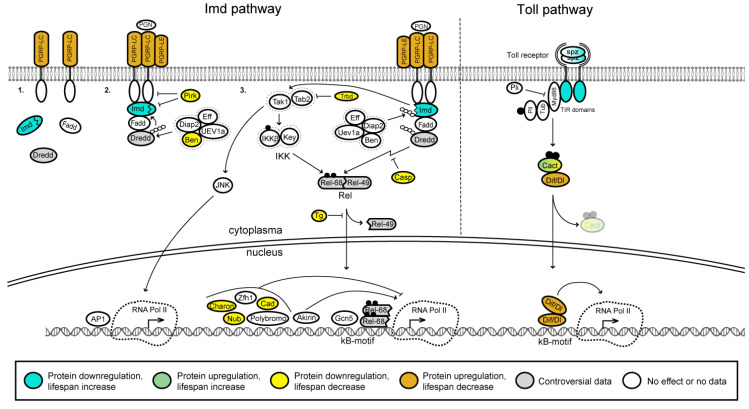
*Drosophila* NF-κB pathway, a simplified schematic representation. The Imd and Toll pathways are shown on the left and right, respectively. Upon infection with Gram-negative bacteria, the Imd pathway is activated through direct recognition of Gram-negative bacterial PGN by PGRP-LC at the cell surface. This creates a transient signaling platform resulting also in the transcription activation of genes encoding AMPs. 1, 2, and 3 indicate successive stages of the Imd pathway activation. In response to both Gram-positive cocci and fungi, Spz (Spatzle) is processed into a biologically active ligand and binds to the Toll receptor. This interaction initiates a proteolytic cascade that results in the transcription activation of genes encoding AMPs. AP-1, activator protein 1 transcription factor; Ben, Bendless (E2 ubiquitin-conjugating enzyme, an Ubc13 homolog); Cact, Cactus; Cad, Caudal; Diap2, Diap protein 2; Dif, transcription factor Dorsal-related immunity factor; Dl, transcription factor Dorsal; Dredd, Death related ced-3/Nedd2-like caspase (also known as Dcp2); Eff, Effete (E2 ubiquitin-conjugating enzyme, an Ubc5 homolog); Fadd, Fas-associated death domain; Gcn5, Gcn5 acetyltransferase (PCAF); IKK, IκB kinase complex; IKKβ, IκB kinase; Imd, Immune deficiency; JNK, c-Jun N-terminal kinase; Key, Kenny; Myd88, adaptor protein Myd88; Nub, Nubbin; Pirk, Poor Imd response upon knock-in; Pli, Pellino (a RING-domain-containing ubiquitin E3 ligase); Pll, Pelle, the serine/threonine kinase ortholog of IRAK; PGN, peptidoglycan; PGRP-LC, Peptidoglycan recognition protein LC; PGRP-LE, Peptidoglycan recognition protein LE; Rel, Relish; Tab2, Tak1-associated binding protein 2; Tak1, (TGF-β)-activating kinase 1; Tg, Transglutaminase; Trbd, Trabid; Tub, adaptor protein Tube; Uev1A, Ubiquitin-conjugating enzyme variant 1A (Ubc/E2 variant (Uev) homolog); Zfh1, Zn finger homeodomain 1. For the other proteins shown in the scheme, the symbols and the full names are identical. Arrows and bar-headed lines indicate activation and inhibition, respectively. Phosphate groups are depicted as solid black dots. Ubiquitin chains are shown as a string of white circles. Protein complexes are encircled by dashed lines. Inactive components of the pathway are shown in semi-transparent mode. Effects of mutations, depletion or overexpression of protein molecules on adult fly lifespan are color-coded according to the legend at the bottom; different simultaneous effects are shown as color gradients (for the references, see Supplementary Table S3).

In different animal species, including *Drosophila* and mammals, aging is associated with the nuclear factor kappa B (NF-κB) signaling pathway [171]. It has been demonstrated that pharmacological inhibition of the NF-κB signaling prevents age-related pathologies and increases the median lifespan of *Drosophila* [171]. Fruit flies possess two NF-κB signaling pathways that are specifically active against different pathogens, the Imd (immune deficiency) signaling activated by most Gram-negative infections and the Toll signaling pathway activated in response to most Gram-positive bacterial and fungal infections [172,173,174] (Figure 4). The three *Drosophila* NF-κB factors—Dorsal (Dl), Dorsal-related immunity factor (Dif) and Relish (Rel)—regulate the insect humoral immunity that gets activated during infection [175,176,177].

Activation of the Imd pathway occurs through direct recognition of Gram-negative bacterial diaminopimelic acid (DAP)-type peptidoglycan (PGN) by the PGN recognition protein LC (PGRP-LC) at the cell surface or by PGRP-LE receptor intracellularly [178,179]. PGRP-LC has several isoforms, including three receptor isoforms (LCx, LCa and LCy). These isoforms have different specificity for extracellular polymeric or monomeric PGN fragments, which are referred to as tracheal cytotoxin (TCT) [180,181,182,183] and trigger the *Drosophila* Imd pathway being presented to PGRP-LE in the cytosol [183]. The PGRP-LB, PGRP-SC1, PGRP-SC2 receptors [184,185] and Pirk protein have been reported to suppress the Imd pathway [186]. Ligand binding causes the receptor to dimerize/multimerize and activates the recruitment of the adaptor proteins Imd and Fadd along with the Death related ced-3/Nedd2-like caspase (Dredd). The initiator caspase Dredd is activated by *Drosophila* inhibitor of apoptosis-2 (DIAP-2)-dependent polyubiquitylation [187]. Activated Dredd cleaves Imd, thus unmasking its domain of interaction with the DIAP-2 [188]. The E3 ubiquitin ligase DIAP-2 associates with cleaved Imd and rapidly conjugates Imd with K63-linked polyubiquitin. K63 polyubiquitin chains are suggested to function as a scaffold to activate transforming growth factor β (TGF-β)-activating kinase 1 (Tak1) and its binding adaptor Tab2 [188,189,190]. The deubiquitinase Trabid (Trbd) negatively regulates the pathway by inactivating Tak1 [190]. The Tak1/Tab2 complex mediates phosphorylation of the IKKβ subunit of the IκB kinase (IKK) on one hand and Jun nuclear kinase (JNK) (see below) on the other [191]. Activated IKKβ, in complex with Kenny (Key), phosphorylates Rel on serines 528 and 529. These phosphorylation sites are not required for Rel cleavage, nuclear translocation, or DNA binding. Instead, they are critical for recruitment of RNA Pol II and antimicrobial peptide (AMP) gene induction. Rel consists of a nuclear factor containing domain (Rel-68) and an inhibitory domain (Rel-49) responsible for keeping Rel in the cytoplasm. Cleavage of the full-length Rel by the caspase Dredd releases the N-terminal domain Rel-68 [192,193,194]. This allows phosphorylated Rel-68 to translocate to the nucleus where it binds as a dimer [195,196,197] to the κB sites within the promoters of genes encoding AMPs (Figure 4).

Phosphorylated Rel-68 forms a complex with PCAF (Gcn5) (histone acetyltransferase (HAT) p300/CBP-associated factor) at target genes and activates transcription [179] (Figure 4). Other regulators such as the transcription factors Caudal [198], Nubbin [199], Zfh1 [200] and Polybromo (or BAP180), a subunit of the chromatin-remodeling SWI/SNF complex [201], keep Rel activity in check, likely by directly antagonizing Rel binding to AMP regulatory DNA in the nucleus [183]. Thus, the activation of the Imd pathway is closely associated with epigenetic regulation and involves the recruitment of chromatin remodeling complexes such as SWI/SNF and HATs, such as PCAF.

Akirin is an NF-κB co-factor required for Imd target gene activation by the Rel transcription factor [202] (Figure 4). H3K4ac, an epigenetic mark of active gene transcription [203], is selectively enriched on Akirin-dependent, but not on Akirin-independent promoters. The presence of Akirin, BAP60 (component of SWI/SNF) and Rel is required at the same level on the promoters for an efficient transcription of Akirin-dependent genes. Akirin may link Rel and BAP60 for recruiting the SWI/SNF complex to the promoters of the Rel target genes [178]. Akirin is required for the transcription activation of direct immune effector (AMP) genes, but not for genes encoding negative regulators of the Imd pathway (except PGRP-SC2) [178]. Thus, Akirin links Rel-dependent Imd signaling to chromatin remodeling by SWI/SNF and this is accompanied by an increase in H3K4ac mark on promoters of Akirin-dependent genes.

Charon (also called Pickle) is a member of the IκB superfamily and inhibits the activity of Rel. Charon fulfills the functional and structural criteria for an IκB protein. It was shown to predominantly reside in the nuclear fraction and regulate the Imd pathway at the level of Rel68:Rel68 homodimer suppressing the Rel68-driven induction of genes encoding AMPs [204] (Figure 4). Caspar (Casp) inhibits Dredd-dependent cleavage of Rel [205]. During infection, it was suggested that certain targets of Rel inhibit JNK signaling through proteasomal degradation of Tak1 in *Drosophila* [206]. Transglutaminase (TG) suppresses the Imd pathway via protein-protein cross-linking that prevents Rel from entering the nucleus [207,208]. Downregulation of TG reduces the lifespan of flies raised under nonsterile conditions but not of flies reared under germ-free conditions [208].

The Toll signaling is activated in response to both Gram-positive cocci and fungi [173]. The pathway is initiated by recognition proteins circulating in the haemolymph, which include the PGN recognition protein SA (PGRP-SA), the Gram-negative binding protein 1 (GNBP1) [209,210]. Upon infection, PGRP-SA and GNBP1 induce a proteolytic cascade that ultimately processes the cytokine-growth factor-like polypeptide Spatzle (Spz, also called Spz-1) into a biologically active ligand, which binds to the ectodomain of Toll-1 receptor [211,212,213,214] (Figure 4). Besides that, *Drosophila* has eight other Toll receptors (Toll-2 to Toll-9) and five other Spz proteins (Spz-2 to Spz-6) [215]. The dimerized activated Toll receptor binds to the adaptor protein Myd88 via intracellular TIR domains [216,217,218]. Upon this interaction, Myd88 recruits Tube (Tub) and the Pelle (Pll) kinase forming the Myd88-Tub-Pll heterotrimeric plasma membrane associated complex [216,219,220] to induce the phosphorylation and subsequent degradation of the IκB inhibitor Cactus (Cact) [221,222]. Cact degradation frees the NF-κB factors Dif and/or Dorsal, which translocate(s) to the nucleus and bind(s) to κB motifs, resulting in transcription activation of genes encoding AMPs [223,224]. It was shown that Dif is the main transcription factor in the innate immunity response in adults and Dorsal is responsible for larvae immune response [224]. Deltex (Dx), a context-dependent regulator of Notch signaling, positively regulates the Toll signaling pathway by facilitating the nuclear localization of the transcription factor(s) Dorsal and/or Dif [225].

**Figure 5:**
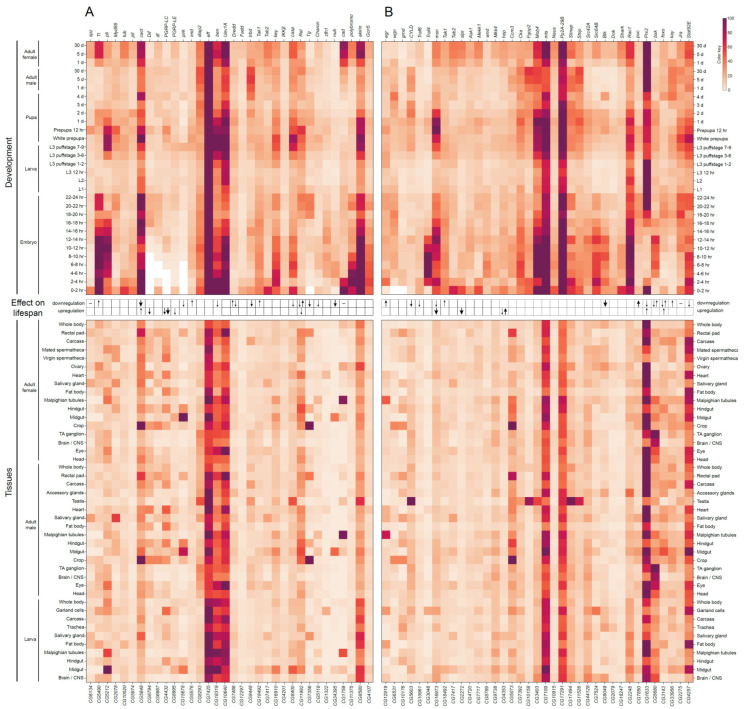
Heat map representation of the expression patterns of genes encoding components of *Drosophila* NF-κB (**A**) and JNK signaling (**B**) pathways during developmental stages (at the top) and across different tissues/organs (at the bottom). Gene expression data were taken from modENCODE and FlyAtlas 2 databases [64,65]. White and deep violet colors represent low and high gene expression levels, respectively. In addition, a summary of effects on fly lifespan caused by gene mutations, ubiquitous or tissue-specific knockdown or overexpression is shown between the gene expression top and bottom heat maps. Up and down arrows indicate increased and decreased adult fly lifespan, respectively; the size of arrows reflects the magnitude of the effect (for the references, see Supplementary Tables S3 and S4).

Tissue-specific and developmental expression profiles of the NF-κB signaling pathway genes are not uniform (Figure 5A and Supplementary Figure S2A). The majority of genes are ubiquitously expressed at moderate levels. Some genes are expressed at very low to moderate levels with minimal variability across developmental stages and different tissues, for example, *imd*, *Dredd*, *Fadd* and *IKKβ* (Figure 5A and Supplementary Figure S2A). Some other genes are generally expressed at low to moderate levels, but at specific developmental stages and tissues they are characterized by relatively high abundance. For instance, the *pirk* gene is very active in midgut of adult flies and larvae, the *Tab2* gene is actively expressed in early embryonic stage (0–2 h), and the *Transglutaminase* (*Tg*) gene is highly expressed in crop of adult flies. As for the IIS and TOR pathways, only a few of the NF-κB pathway genes are active in all tissues and at all stages of development: *effete* (*eff*), *bendless* (*ben*) and *Ubiquitin-conjugating enzyme variant 1A* (*Uev1A*). Ben and Uev1A form a heterodimer that is required for Imd K63-linked polyubiquitinylation and its subsequent proteasome degradation [226,227,228]. Eff is also required for Imd ubiquitinylation and regulation of cell apoptosis [188]. Thus, it appears that in *Drosophila*, the Gram-positive bacterial response system that is triggered via the Imd pathway is constitutively ready to activate antimicrobial response genes. This may also have an adaptive value, and a decrease in the readiness of this system to fight infections due to mutations that disrupt the expression of its components, leads to a corresponding decrease in lifespan.

Downregulation of some components of the NF-κB signaling pathway was shown to slightly extend *Drosophila* lifespan (Supplementary Table S3). For instance, downregulation of the *Toll* (*Tl*) gene extends lifespan for about 12% and 2% in females and males, respectively [229]. Downregulation of *imd* leads to about 15% and 12% increase of mean lifespan in heterozygous male and female flies [230]. Downregulation of the *cact*, *pirk*, *ben*, *trbd*, *casp*, *Tg*, *Charon* and *nubbin (nub*) genes were demonstrated to cause moderate decrease of *Drosophila* lifespan (Figure 5A, Supplementary Table S3) [173,190,191,229,231,232,233]. The data on the influence of the *Dredd* and *Rel* mutations on fruit fly lifespan are controversial. *Dredd* mutation leads to 8–12% increase of the lifespan compared to control [191]. At the same time RNAi of the *Dredd* gene was found to cause 10 days increase and 15 days decrease of lifespan compared to control in male and female flies, respectively [234]. *Rel*-null flies were shown to have a statistically significant reduction (for about 23%) in the lifespan compared with control, but RNAi of *Rel* in glia, neurons and intestine resulted in about 60%, 38% and 35% increase of the lifespan, respectively [191].

Upregulation of the *cact* gene in neuroblasts increases the median lifespan of male and female flies for about 16% and 4%, respectively [173]. Overexpression of other genes (e.g., *Dif*, *PGRP-LC*, *PGRP-LE*, *Rel*) negatively regulates fly lifespan. Finally, shortened lifespan was observed in *Dif*, *PGRP-LC*, *PGRP-LE* and *Rel* mutants [173,212,235,236] (Figure 5A, Supplementary Table S3).

### 3.4. JNK Signaling Pathway

The c-Jun NH_2_-terminal kinase (JNK) is a conserved branch of the mitogen-activated protein kinase (MAPK) pathway [237]. JNK signaling is activated by cytokines and environmental stress [238] and regulates a number of cellular processes such as cellular proliferation, migration and apoptosis [239]. In *Drosophila*, the JNK signaling can be loosely classified into “canonical” and “non-canonical” pathways. The term ‘‘canonical’’ is used to describe pathway components activated during development, especially during embryonic dorsal closure, which are also required for normal wound closure [240,241]. “Non-canonical” signaling pathway is not required for development and is activated during stresses and wound healing [240,241].

**Figure 6:**
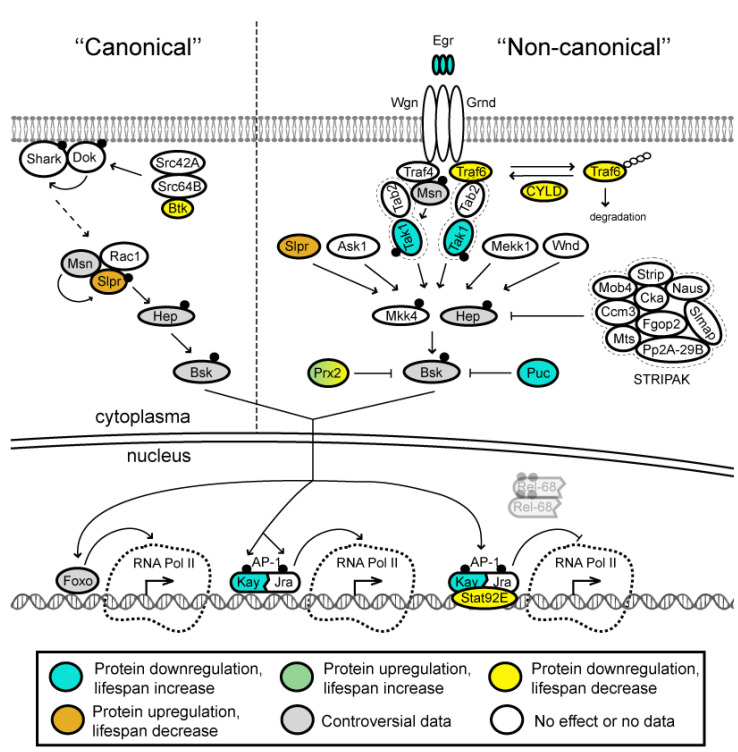
The JNK pathway in *Drosophila*, a simplified schematic representation. Stress-induced JNK pathway is activated through Egr interaction with receptors Wgn or Grnd. This interaction initiates the hierarchical phosphorylation and subsequent activation of the MAPK cascade. AP-1, activator protein 1 transcription factor; Ask1, Apoptotic signal-regulating kinase 1; Bsk, Basket; Btk, Bruton tyrosine kinase; Ccm3, Cerebral cavernous malformation 3; Cka, Connector of kinase to AP-1; CYLD, Cylindromatosis; Dok, Downstream of kinase; Egr, Eiger; Fgop2, Fibroblast growth factor receptor 1 oncogene partner 2; Foxo, Forkhead box, sub-group O; Grnd, Grindelwald; Hep, Hemipterous; Jra, Jun-related antigen; Kay, Kayak; Mkk4, MAP kinase kinase 4; Mob4, MOB kinase activator 4; Msn, Misshapen; Naus, Nausicaa; Pp2A-29B, Protein phosphatase 2A at 29B; Prx2, Peroxiredoxin 2; Puc, Puckered; Shark, SH2 domain ankyrin repeat kinase; Slmap, Sarcolemma associated protein; Slpr, Slipper; Src42A, Src oncogene at 42A; Src64B, Src oncogene at 64B; Stat92E, Signal-transducer and activator of transcription protein at 92E; STRIPAK, Striatin-interacting phosphatase and kinase complex; Strip, Striatin interacting protein; Tab2, Tak1-associated binding protein 2; Tak1, TGF-β activated kinase 1; Traf4, TNF-receptor-associated factor 4; Traf6, TNF-receptor-associated factor 6; Wgn, Wengen; Wnd, Wallenda. Arrows and bar-headed lines indicate activation and inhibition, respectively. Phosphate groups are depicted as solid black dots. Ubiquitin chain is shown as a string of white circles. Protein complexes are encircled by dashed lines. Effects of mutations, depletion or overexpression of protein molecules on adult fly lifespan are color-coded according to the legend at the bottom; different simultaneous effects are shown as color gradients (for the references, see Supplementary Table S4).

“Non-canonical” signaling is activated by binding of the single tumor necrosis factor (TNF) ligand Eiger (Egr) to the TNF receptors Wengen (Wgn) and Grindelwald (Grnd) [242,243,244,245,246] that have been implicated in different cellular pathways [245] (Figure 6). Each TNF receptor forms hetero-hexamers with trimeric Egr ligands, although with different affinities [245]. Egr/Grnd activity is transduced via TNF receptor associated factor (TRAF) proteins and results in the activation of the JNK pathway [247]. *Drosophila* has only two TRAF proteins, Traf4 and Traf6 [248,249]. Originally, Traf4 was suggested to be the adaptor protein that links Wgn and the JNK cascade [250,251], since only Traf4, but not Traf6 binds to Mishappen (Msn), the first kinase in the JNK pathway [249]. However, further biochemical and genetic studies have revealed that Traf6, but not Traf4 plays this role [250,252,253,254]. Additionally, it was found that TRAF proteins transduce discrete signaling pathways in *Drosophila*. Overexpression of Traf4 causes JNK pathway activation, while overexpression of Traf6 leads to activation of *Drosophila* NF-κB proteins Dif and Rel [255]. In addition, deubiquitinating enzyme CYLD regulates JNK activation by deubiquitinating Traf6, thus, preventing it from ubiquitin-mediated proteolytic degradation in cytoplasma [252].

Ligand-receptor binding initiates a conserved signaling cascade of MAPK superfamily kinases, which includes at least five JNKK kinases (JNKKKs)—Slipper (Slpr), Wallenda (Wnd), Tak1, Mekk1 and Apoptotic signal-regulating kinase 1 (Ask1) as well as at least two JNK kinases (JNKKs)—Hemipterous (Hep) and MAP kinase kinase 4 (Mkk4) [256,257,258,259,260,261]. The ultimate goal of this JNK cascade is activation of the c-Jun N-terminal kinase Basket (Bsk) (Figure 6), which results in a variety of tissue-specific and context-specific cellular responses [243,262].

Activated (phosphorylated) Bsk translocates into the nucleus and, in its turn, activates its downstream transcription factor Jun-related antigen (Jra), which heterodimerizes with the transcription factor Kayak (Kay) forming an active AP-1 transcriptional complex in the nucleus [254,263] (Figure 6). AP-1 transcriptionally upregulates numerous target genes and AP-1 dimers bind to different types of palindromic sequences. Thus, Kay:Jun and Jun:Jun dimers preferentially bind to DNA motifs referred to as tetradeconyl phorbol acetate-responsive elements (TREs, also called AP-1 motifs) and, with slightly lower affinity, to cyclic adenosine monophosphate-responsive elements (CREs). One well-established AP-1 target gene is *puckered* (*puc*) [264] encoding a phosphatase that dephosphorylates Bsk in an induced-negative feedback loop (Figure 6). Other targets include the *unpaired* (*upd*) gene encoding the *Drosophila* interleukin-6 (IL-6) homolog [265] and the *Matrix metalloproteinase 1* (*Mmp1*) gene encoding a protease that degrades the extracellular matrix [266]. AP-1 also forms a repressosome complex together with the Stat92E protein. This complex blocks the expression of immune effector genes (such as those encoding AMPs) by competitively blocking Rel binding and by recruiting histone deacetylase at their promoters [2,267].

Activation of the “canonical” and “non-canonical” JNK signaling pathways differs only upstream, but not downstream of the Hep component. The most upstream acting molecules identified for the “canonical” signaling during dorsal closure are nonreceptor tyrosine kinases of the Src family: Src42A, Src64B and Btk [240] (Figure 6). Src42A phosphorylates Downstream of kinase (Dok), which belongs to the Dok family of adaptor proteins. Dok is required for the appropriate tyrosine phosphorylation, membrane localization and activation of nonreceptor tyrosine kinase Shark [268]. How these kinases relay the signal to downstream components is still not well understood. However, it was shown that a serine threonine kinase Slipper (Slpr) interacts physically with Rac1 and with Msn, the latter of which is supposed to phosphorylate Slpr, thus activating it. Activated Slpr in its turn phosphorylates Hep. Then, the “canonical” and “non-canonical” pathways intersect [240,269].

It is also known that JNK antagonizes IIS, since JNK signaling promotes Foxo nuclear localization [270] (Figure 6). Foxo also regulates activity of numerous genes (see above). Among Foxo targets are cytoprotective genes such as *Peroxiredoxin* (*Prx2*), *lethal (2) essential for life* (*l(2)efl*) and *Thor* (eIF4E-binding protein, an inhibitor of cap-dependent protein synthesis), which activity advances stress defense [270,271]. Overexpression of *Prx2* is able to suppress Bsk activity in neurons, indicating that Prx2 may also act as a negative feedback regulator of JNK in specific tissues [271].

Along with *Puc* and *Prx2* inhibition, JNK signaling is downregulated by the STRIPAK complex components, Striatin interacting protein (Strip) and Connector of kinase to AP-1 (Cka), which bind to and inactivate Hep/JNKK acting as negative regulators of JNK signaling [272,273,274,275,276,277].

The JNK pathway is activated not only through Wgn and Grnd receptors but also from the Imd pathway through Tak1 (Figure 4 and Figure 6) [206,278]. JNK-regulated lipopolysaccharides-responsive genes exhibit transient expression, whereas IKK/Rel target genes demonstrate sustained expression pattern. Rel-dependent genes not only serve their own immune effector roles, but also participate in shaping the temporal pattern of the JNK-mediated immune response by inactivating Tak1. Thus, IKK/Rel branch plays an active role in turning off JNK activity [206].

Tissue-specific and developmental expression profiles of the JNK signaling pathway genes are not uniform (Figure 5B and Supplementary Figure S2B). Most genes of the pathway are expressed at moderate levels. The *grnd*, *Traf6*, *wnd*, *Ask1*, *hep* and *Mkk4* genes are expressed at very low to moderate levels with minimal variability across developmental stages and different tissues (Figure 5B and Supplementary Figure S2B). The *CYLD*, *Fgop2*, *Slmap* and *Strip* genes are generally expressed at low levels, but they are very active in testis of adult males. The *egr* gene is active in Malpighian tubules of adult flies, while *bsk* expression first appears in CNS close to the end of the pupal stage and then it is kept active in the head, eye, brain and thoracic ganglion of adult flies. As with the other pathways we have described here, IIS, TOR and NF-κB, several genes of the JNK pathway (e.g., *mts*, *Pp2A-29B*, *Prx2*) are highly upregulated in virtually all tissues, and the corresponding products of these genes were found almost at all developmental stages (Figure 5B and Supplementary Figure S2B). The subunits of the PP2A phosphatase, Mts and Pp2A-29B (catalytic and scaffold subunit, respectively), which suppress the activity of the IIS pathway due to dephosphorylation of the Akt kinase, together with the Wdb regulatory subunit and Prx2 inhibitor appear to reliably inhibit the JNK cascade. This inhibition seems to be supported by the weak expression of the proteins of the STRIPAK complex, of which only Cka is expressed at an average level. At the same time, several main members of the JNK cascade, the kinases Msn, Mkk4 and Bsk, are ubiquitously expressed at moderate level. Thus, the JNK pathway is most likely inhibited in cells by default, but it is at the ready because all the kinases required for Egr signaling are constitutively present.

Downregulation of several components of the JNK signaling pathway (e.g., mutations of the *egr*, *Tak1*, *kay* and *puc* genes) slightly extend *Drosophila* lifespan (Figure 5B, Supplementary Table S4). On the contrary, mutations of the *CYLD*, *Traf6*, and *Prx2* genes lead to the opposite effect on the *Drosophila* lifespan, causing its reduction. Upregulation of the *slpr* gene results in highly shortened lifespan of flies. At the same time, upregulation of the *foxo* and *Prx2* genes causes slight extension of fly longevity (Figure 5B, Supplementary Table S4). It is rather difficult to understand the mechanisms of influence of the JNK pathway on lifespan, since this pathway has pleiotropic effects: it maintains stem cell renewal in homeostasis, induces apoptosis in case of damage, induces proliferation accompanied by switching the type of cell divisions and reprogramming of cells during regeneration [237]. However, the most significant increase in lifespan was observed with inhibition of the JNK pathway. Particularly, mutations of the *egr* gene, overexpression of Puc, and overexpression of the transcription factor Foxo gave the greatest increase in lifespan up to 62% (Supplementary Table S4). Moreover, Foxo, inducing genes associated with the suppression of proliferation and protein synthesis also induced the expression of another inhibitor of the JNK pathway, Prx2 [271]. Thus, although mutations and upregulation of some components of the JNK pathway lead to a sharp decrease in lifespan, suppression of signaling through the JNK pathway is generally associated with increased lifespan.

### 3.5. Interconnection between IIS, TOR, NF-κB and JNK Pathways

The interactions between the above-described pathways are much better studied in mammals. Signaling through the human IIS pathway involves kinases such as p38 (MAPK), JNK, AMPK, PKC and others and most of these kinases are able to phosphorylate several key regulators of glucose homeostasis as well as the insulin receptor, resulting in decreased activity of the PI3K/Akt [279]. Besides, it was shown that JNK antagonizes IIS, promoting FOXO nuclear localization [270]. This is how the interactions between the JNK, NF-κB and the IIS pathways can take place.

In *Drosophila*, the interactions between the pathways are rather poorly understood. The IIS pathway, as the central pathway associated with nutrient intake, interacts with all other pathways and plays an important role not only in the regulation of longevity, but also in the development of cancer in *Drosophila* [280]. As for the interaction between the IIS and the TOR pathways, it is obvious that the cap-dependent protein synthesis depends on the presence of amino acids and carbohydrates, and a decrease in their intake suppresses TOR, turns off protein synthesis, and reduces overall metabolism. This, in turn, leads to some extension of lifespan. An approach used by many investigators to reduce the activity of the insulin/TOR pathway is DR [3].

With regard to the the NF-κB and the JNK pathways, both of them appear to be involved in the immune response in *Drosophila* and are interconnected through the Tak1 kinase [206,227,278]. Activation of Rel seems to be associated with proteasomal degradation of Tak1 required for JNK signaling [206]. In turn, the degradation of Tak1 leads to the cessation of JNK signaling within 1 h. Thus, in the case of immune activation, a transient JNK and long-term NF-κB responses occur. The same relationship between the two pathways is observed in humans [206]. Interestingly, JNK pathway is not required for Rel activation, but is absolutely necessary for the immune response and production of antimicrobial peptides in *Drosophila* [278]. Recently, the role of the immune signaling in the fly brain and its association with longevity and neurodegenerative diseases has been demonstrated, placing the nervous system as the master system controlling lifespan [173,191]. The relationship between the immune and nervous systems in humans has also been discussed lately [281,282].

## 4. Concluding Remarks

Evolutionary conserved signaling pathways IIS, TOR, NF-κB and JNK play critical roles under stress conditions and aging. In *Drosophila*, each pathway consists of the numerous components, which are characterized by a larger number of paralogous subunits in human (Supplementary Tables S1–S4). This indicates the importance of these pathways and their development in evolution from a common ancestor of invertebrates and vertebrates.

In *Drosophila*, activation of the IIS and the TOR pathways leads to a shortening of lifespan, whereas activation of the JNK pathway suppresses the activity of TOR and causes an increase in lifespan. Violation of the NF-κB pathway results in a decrease in lifespan. The Imd sub-branch of the NF-κB pathway is constitutively ready for activation and both activating and repressing components of the IIS and the TOR pathways are constitutively expressed.

In closing, many questions regarding the links between the components of these signaling pathways and aging still remain. For instance, influence of downregulation or overexpression of genes on fly lifespan was studied only for 40–53% of the pathways’ components (Figure 2 and Figure 5). Moreover, some of these data seem to be controversial. Among constitutively expressed subunits of the pathways, effect on lifespan had been so far demonstrated only for 25% of them. In the future, it will be important to detect the pathways’ components with the highest magnitude of the effect on lifespan resulted from their misexpression.

## Supplementary Materials

The following supporting information can be downloaded at: https://www.mdpi.com/article/10.3390/ijms231911244/s1. References [283,284,285,286,287,288,289,290,291,292,293,294,295,296,297,298,299,300,301] are cited in the Supplementary Materials.
