# Supplementary material for: Drosophila as a Model Organism to Study Basic Mechanisms of Longevity
Source: Int J Mol Sci. 2022 Sep 24;23(19):11244. doi: 10.3390/ijms231911244 (PMC9569508; doi:10.3390/ijms231911244)
Supplement: Supplementary file 1 [file ijms-23-11244-s001.zip › Supplementary Table S1.pdf]

Supplementary Table S1. Summary of input signals and chromatin effector proteins for IIS signaling pathway controlling *Drosophila* lifespan. *Drosophila* and human protein symbols are provided according to the FlyBase annotation (March 29, 2022; http://flybase.org/). Alternative protein symbols (synonyms) are indicated within brackets.

Components	Human orthologs	Effect(s) on lifespan	Reference(s)
**Ilp1**<br>**Insulin-like peptide 1**<br>(CG14173)	IGF1<br>IGF2	-	-
**Ilp2**<br>**Insulin-like peptide 2**<br>(CG8167)	IGF1<br>IGF2	Knockout of *Ilp2* results in a median adult lifespan extension of 9% and 13% for males and females, respectively.<br><br>Partial genetic ablation of the neurosecretory Ilp-producing cells that reside in the brain and are the primary producers of Ilp2, Ilp3 and Ilp5 in the adult body resulted in median lifespan increase by 10.5% in males and 18.5% and 33.5% in virgin and mated females, respectively.	[1]<br><br>[2]
**Ilp3**<br>**Insulin-like peptide 3**<br>(CG14167)	IGF1<br>IGF2<br>INS	Partial genetic ablation of the neurosecretory Ilp-producing cells that reside in the brain and are the primary producers of Ilp2, Ilp3 and Ilp5 in the adult body resulted in median lifespan increase by 10.5% in males and 18.5% and 33.5% in virgin and mated females, respectively.	[2]
**Ilp4**<br>**Insulin-like peptide 4**<br>(CG6736)	INSL3	-	-
**Ilp5**<br>**Insulin-like peptide 5**<br>(CG33273)	INS<br>INS-IGF2	Partial genetic ablation of the neurosecretory Ilp-producing cells that reside in the brain and are the primary producers of Ilp2, Ilp3 and Ilp5 in the adult body resulted in median lifespan increase by 10.5% in males and 18.5% and 33.5% in virgin and mated females, respectively.	[2]
**Ilp6**<br>**Insulin-like peptide 6**<br>(CG14049)	IGF1<br>IGF2	-	-
**Ilp7**<br>**Insulin-like peptide 7**<br>(CG13317)	-	-	-
**InR**<br>**Insulin-like receptor**<br>(CG18402)	IGF1R<br>INSR<br>INSRR<br>LMTK2	Females of transheterozygous *InR* flies are 85% longer lived than wild-type controls. Other homo- and transheterozygous *InR* mutants were found to be strongly or moderately short-lived.<br><br>The heterozygote $InR^{E19(HR)}/InR^{+(HR)}$ does not extend lifespan. Other transheterozygous and hemizygous *InR* mutants strongly or moderately extended lifespan.<br><br>There was no difference in lifespan between flies	[3]<br><br>[4]<br><br>[5]

		with cardiac-specific overexpression of *InR* and flies with cardiac-specific overexpression of *Pten*.<br><br>Activation of InR expression in intestinal stem cells and enteroblasts results in 6% and 10% reduction of mean lifespan in males and females, respectively, compared with control. Induced inhibition of InR in intestine using dominant-negative InR causes 7% extension of mean lifespan compared with uninduced flies.	 <br><br>[6]
**Chico**<br>(CG5686)	IRS1<br>IRS2<br>IRS4	Heterozygous *chico* males and females showed increase of median adult lifespan of 13% and 36%, respectively. Homozygous *chico* females exhibited an increase of median and maximum lifespan of up to 48% and 41%, respectively.<br><br>Heterozygous *chico* males and females showed increase of lifespan of 51% and 37%, respectively. Homozygous *chico* females and males showed increase of lifespan of 59% and 6%, respectively.	[7]<br><br><br><br><br><br>[8]
**Lnk**<br>(CG17367)	SH2B2<br>SH2B1<br>SH2B3	Heterozygous *Lnk* flies did not show any significant differences in lifespan. Homozygous *Lnk* females and males showed increase of median lifespan of approximately 11% and 15%, respectively.	[9]
**Pi3K21B**<br>(CG2699)	PIK3R3<br>PIK3R2<br>PIK3R1<br>LOC110117<br>498-PIK3R3	-	-
**Pi3K92E**<br>**Phosphatidylinositol 3-kinase 92E**<br>(CG4141)	PIK3CD<br>PIK3CB<br>PIK3CA<br>PIK3CG	Overexpression of dominant-negative Pi3K92E in intestinal stem cells and enteroblasts results in 3% and 5% reduction of mean lifespan in males and females, respectively, compared with control. Induced inhibition of Pi3K92E in intestine using dominant-negative Pi3K92E causes 7% extension of mean lifespan compared with uninduced flies.	[6]
**Pten**<br>**Phosphatase and tensin homolog**<br>(CG5671)	PTEN<br>TPTE2<br>TPTE	*Pten* overexpression in adult head fat body increased the median lifespan of male and female flies of 19% and 17%, respectively. *Pten* overexpression in fat body had no significant effect on lifespan.<br><br>*Pten* overexpression in muscles extends the median lifespan of males of 20%.<br><br>There was no difference in lifespan between flies with cardiac-specific overexpression of *InR* and flies with cardiac-specific overexpression of *Pten*.	[10]<br><br><br><br><br><br>[11]<br><br><br>[5]
**Pdk1**<br>**Phosphoinositide-dependent kinase 1**<br>(CG1210)	PDPK1<br>PDPK2P	-	-
**Akt**<br>**Akt kinase**<br>(CG4006)	AKT2<br>AKT3<br>AKT1<br>SGK1	Inhibition of Akt expression in intestinal stem cells and enteroblasts results in 11% and 7% reduction of mean lifespan in males and females, respectively, compared with control. Induced	[6]

	SGK2<br>C8orf44-SGK3	inhibition of Akt in intestine using RNAi causes more than 10% extension of mean lifespan compared with uninduced flies.	
**Foxo**<br>**Forkhead box, sub-group O**<br>(CG3143)	FOXO3<br>FOXO1<br>FOXO4	Heterozygous *foxo* males and females showed decrease of mean lifespan of about 5% and increase of mean lifespan of 29%, respectively.<br><br>Overexpression of *foxo* in adult head fat body increased the median lifespan of male and female flies of about 24% and 16%, respectively. Pan-neuronal overexpresion of *foxo* as well as its overexpression in fat body, neurolemma and glial cells had no significant effect on lifespan.<br><br>Overexpression of *foxo* in muscles extends the median lifespan of male flies of about 23%.<br><br>Induced expression of *foxo* in the adult fat body increased lifespan of female flies by 20 to 50%.<br><br>Heterozygosity for $foxo^{21}$ or $foxo^{25}$ does not affect life span in wild-type backgrounds.<br><br>Activation of Foxo expression in intestinal stem cells and enteroblasts results in 6% and 4% reduction of mean lifespan in males and females, respectively, compared with control.	[12]<br><br>[10]<br><br>[11]<br><br>[13]<br><br>[14]<br><br>[6]
**14-3-3ε**<br>(CG31196)	YWHAE	Heterozygous *14-3-3ε* males and females showed increase of mean lifespan of about 61% and 21%, respectively.	[12]
**14-3-3ζ**<br>(CG17870)	YWHAZ<br>YWHAB<br>YWHAG<br>YWHAH<br>SFN<br>YWHAQ	-	-

References

1. Grönke, S.; Clarke, D.F.; Broughton, S.; Andrews, T.D.; Partridge, L. Molecular evolution and functional characterization of Drosophila insulin-like peptides. *PLoS Genet* **2010**, *6*, e1000857, doi:10.1371/journal.pgen.1000857.
2. Broughton, S.J.; Piper, M.D.; Ikeya, T.; Bass, T.M.; Jacobson, J.; Driege, Y.; Martinez, P.; Hafen, E.; Withers, D.J.; Leevers, S.J.; et al. Longer lifespan, altered metabolism, and stress resistance in Drosophila from ablation of cells making insulin-like ligands. *Proc Natl Acad Sci U S A* **2005**, *102*, 3105-3110, doi:10.1073/pnas.0405775102.
3. Tatar, M.; Kopelman, A.; Epstein, D.; Tu, M.P.; Yin, C.M.; Garofalo, R.S. A mutant Drosophila insulin receptor homolog that extends life-span and impairs neuroendocrine function. *Science* **2001**, *292*, 107-110, doi:10.1126/science.1057987.
4. Yamamoto, R.; Palmer, M.; Koski, H.; Curtis-Joseph, N.; Tatar, M. Aging modulated by the Drosophila insulin receptor through distinct structure-defined mechanisms. *Genetics* **2021**, *217*, doi:10.1093/genetics/iyaa037.
5. Wessells, R.J.; Fitzgerald, E.; Cypser, J.R.; Tatar, M.; Bodmer, R. Insulin regulation of heart function in aging fruit flies. *Nat Genet* **2004**, *36*, 1275-1281, doi:10.1038/ng1476.

6. Biteau, B.; Karpac, J.; Supoyo, S.; Degennaro, M.; Lehmann, R.; Jasper, H. Lifespan extension by preserving proliferative homeostasis in Drosophila. *PLoS Genet* **2010**, *6*, e1001159, doi:10.1371/journal.pgen.1001159.
7. Clancy, D.J.; Gems, D.; Harshman, L.G.; Oldham, S.; Stocker, H.; Hafen, E.; Leevers, S.J.; Partridge, L. Extension of life-span by loss of CHICO, a Drosophila insulin receptor substrate protein. *Science* **2001**, *292*, 104-106, doi:10.1126/science.1057991.
8. Tu, M.P.; Epstein, D.; Tatar, M. The demography of slow aging in male and female Drosophila mutant for the insulin-receptor substrate homologue chico. *Aging Cell* **2002**, *1*, 75-80, doi:10.1046/j.1474-9728.2002.00010.x.
9. Slack, C.; Werz, C.; Wieser, D.; Alic, N.; Foley, A.; Stocker, H.; Withers, D.J.; Thornton, J.M.; Hafen, E.; Partridge, L. Regulation of lifespan, metabolism, and stress responses by the Drosophila SH2B protein, Lnk. *PLoS Genet* **2010**, *6*, e1000881, doi:10.1371/journal.pgen.1000881.
10. Hwangbo, D.S.; Gershman, B.; Gersham, B.; Tu, M.P.; Palmer, M.; Tatar, M. Drosophila dFOXO controls lifespan and regulates insulin signalling in brain and fat body. *Nature* **2004**, *429*, 562-566, doi:10.1038/nature02549.
11. Demontis, F.; Perrimon, N. FOXO/4E-BP signaling in Drosophila muscles regulates organism-wide proteostasis during aging. *Cell* **2010**, *143*, 813-825, doi:10.1016/j.cell.2010.10.007.
12. Nielsen, M.D.; Luo, X.; Biteau, B.; Syverson, K.; Jasper, H. 14-3-3 Epsilon antagonizes FoxO to control growth, apoptosis and longevity in Drosophila. *Aging Cell* **2008**, *7*, 688-699, doi:10.1111/j.1474-9726.2008.00420.x.
13. Giannakou, M.E.; Goss, M.; Jünger, M.A.; Hafen, E.; Leevers, S.J.; Partridge, L. Long-lived Drosophila with overexpressed dFOXO in adult fat body. *Science* **2004**, *305*, 361, doi:10.1126/science.1098219.
14. Wang, M.C.; Bohmann, D.; Jasper, H. JNK extends life span and limits growth by antagonizing cellular and organism-wide responses to insulin signaling. *Cell* **2005**, *121*, 115-125, doi:10.1016/j.cell.2005.02.030.
