# Supplementary material for: Drosophila as a Model Organism to Study Basic Mechanisms of Longevity
Source: Int J Mol Sci. 2022 Sep 24;23(19):11244. doi: 10.3390/ijms231911244 (PMC9569508; doi:10.3390/ijms231911244)
Supplement: Supplementary file 1 [file ijms-23-11244-s001.zip › Supplementary Table S2.pdf]

Supplementary Table S2. Summary of input signals and chromatin effector proteins for TOR signaling pathway controlling *Drosophila* lifespan. *Drosophila* and human protein symbols are provided according to the FlyBase annotation (March 29, 2022; http://flybase.org/). Alternative protein symbols (synonyms) are indicated within brackets.

**Components**		**Human orthologs**	**Effect(s) on lifespan**	**Reference(s)**
**Slif**<br>**Slimfast**<br>(CG11128)		SLC7A1<br>SLC7A3<br>SLC7A2	-	-
**Sesn**<br>**Sestrin**<br>(CG11299)		SESN3<br>SESN1<br>SESN2	*Sesn* mutant flies have median lifespan reduced by 13% on the 1.0× yeast food, compared with controls. Ubiquitous overexpression of *Sesn* significantly increased lifespan with a median extension of 10%. Overexpression of *Sesn* in neurons, fat bodies and enterocytes did not cause lifespan extension. In contrast, overexpression of *Sesn* in intestinal stem cells resulted in a significant lifespan extension, again with a median lifespan extension of ~10%.	[1]
**GATOR2 complex**	**Mio**<br>**Missing oocyte**<br>(CG7074)	MIOS	-	-
	**Nup44A**<br>**Nucleoporin at 44A**<br>(CG8722)	SEH1L	-	-
	**Sec13**<br>**Secretory 13**<br>(CG6773)	SEC13	-	-
	**Wdr24**<br>**WD repeat domain 24**<br>(CG7609)	WDR24	-	-
	**Wdr59**<br>**WD repeat domain 59**<br>(CG4705)	WDR59	-	-
**GATOR1 complex**	**Iml1**<br>**Increased minichromosome loss 1**<br>(CG12090)	DEPDC5	-	-
	**Nprl2**<br>**Nitrogen permease regulator-like 2**<br>(CG9104)	NPRL2	The median lifespan of *Nprl2* null mutants (34 d) was markedly shorter than that of wild-type flies (64 d).	[2]
	**Nprl3**<br>**Nitrogen permease regulator-like 3**<br>(CG8783)	NPRL3	-	-
**RAGULATOR**	**Lamtor1**<br>**Late**	LAMTOR1	-	-

**complex**	**endosomal/lysosomal adaptor, MAPK and MTOR activator 1** (CG14184)			
	**Lamtor2 Late endosomal/lysosomal adaptor, MAPK and MTOR activator 2** (CG5189)	LAMTOR2	-	-
	**Lamtor3 Late endosomal/lysosomal adaptor, MAPK and MTOR activator 3** (CG5110)	LAMTOR3	-	-
	**Lamtor4 Late endosomal/lysosomal adaptor, MAPK and MTOR activator 4** (CG14977)	LAMTOR4	-	-
	**Lamtor5 Late endosomal/lysosomal adaptor, MAPK and MTOR activator 5** (CG14977)	LAMTOR5	-	-
**VACUOLAR ATPASE SUBUNITS** (FBgg0000111)	**ATP6AP2 ATPase H+ transporting accessory protein 2** (CG8444)	ATP6AP2	-	-
	**CG5421**	ATP6AP1 ATP6AP1L	-	-
	**VhaAC45 Vacuolar H+ ATPase AC45 accessory subunit** (CG8029)	ATP6AP1 ATP6AP1L	-	-

	**VhaAC45RP VhaAC45-related protein** (CG31030)	ATP6AP1<br>ATP6AP1L	Knock-down of *VhaAC45RP* expression in neurons decreases adult longevity. Pan-neuronal expression of *VhaAC45RP*^{RNAi3} and Dcr-2 led to a marked shortening of the lifespan of adult flies.	[3]
	**Vha16-1 Vacuolar H+ ATPase 16kD subunit 1** (CG3161)	ATP6V0C	-	-
	**Vha16-2 Vacuolar H+ ATPase 16kD subunit 2** (CG32089)	ATP6V0C	-	-
	**Vha16-3 Vacuolar H+ ATPase 16kD subunit 3** (CG32090)	ATP6V0C	-	-
	**Vha16-4 Vacuolar H+ ATPase 16kD subunit 4** (CG9013)	ATP6V0C	-	-
	**Vha16-5 Vacuolar H+ ATPase 16kD subunit 5** (CG9013)	ATP6V0C	-	-
	**Vha100-1 Vacuolar H+ ATPase 100kD subunit 1** (CG1709)	ATP6V0A1<br>ATP6V0A4<br>ATP6V0A2<br>MIR5010	-	-
	**Vha100-2 Vacuolar H+ ATPase 100kD subunit 2** (CG18617)	ATP6V0A4<br>ATP6V0A1<br>ATP6V0A2	-	-
	**Vha100-3 Vacuolar H+ ATPase 100kD subunit 3** (CG30329)	ATP6V0A1<br>ATP6V0A2<br>ATP6V0A4<br>TCIRG1	-	-
	**Vha100-4 Vacuolar H+ ATPase**	ATP6V0A4<br>ATP6V0A2<br>TCIRG1	-	-

	**100kD subunit 4** (CG7678)			
	**Vha100-5 Vacuolar H+ ATPase 100kD subunit 5** (CG12602)	ATP6V0A1 ATP6V0A4 ATP6V0A2	-	-
	**VhaAC39-1 Vacuolar H+ ATPase AC39 subunit 1** (CG2934)	ATP6V0D1 ATP6V0D2	Gut-specific and systemic *VhaAC39-1* RNAi lines showed the shortening of median lifespan of flies by 63% and 9%, respectively, in comparison with the control. Oerexpression of *VhaAC39-1* did not affect the longevity of flies. Additionally, a lifespan assay in an independent *VhaAC39-1* RNAi line showed that the median lifespan of gut-specific and systemic *VhaAC39-1* RNAi flies was shortened by 68% and 49%, respectively.	[4]
	**VhaAC39-2 Vacuolar H+ ATPase AC39 subunit 2** (CG4624)	ATP6V0D1 ATP6V0D2	-	-
	**VhaM9.7-a Vacuolar H+ ATPase M9.7 subunit a** (CG1268)	ATP6V0E1 ATP6V0E2	-	-
	**VhaM9.7-b Vacuolar H+ ATPase M9.7 subunit b** (CG7625)	ATP6V0E1 ATP6V0E2	-	-
	**VhaM9.7-c Vacuolar H+ ATPase M9.7 subunit c** (CG11589)	ATP6V0E1 ATP6V0E2	-	-
	**VhaM9.7-d Vacuolar H+ ATPase M9.7 subunit d** (CG14909)	ATP6V0E1 ATP6V0E2	-	-
	**VhaPPA1-1 Vacuolar H+ ATPase PPA1 subunit 1** (CG7007)	ATP6V0B	-	-
	**VhaPPA1-2 Vacuolar H+ ATPase PPA1**	ATP6V0B	-	-

	**subunit 2** (CG7026)			
	**Vha68-1 Vacuolar H+ ATPase 68kD subunit 1** (CG12403)	ATP6V1A	-	-
	**Vha68-2 Vacuolar H+ ATPase 68kD subunit 2** (CG3762)	ATP6V1A	-	-
	**Vha68-3 Vacuolar H+ ATPase 68kD subunit 3** (CG5075)	ATP6V1A	-	-
	CG15719	ATP6V1F	-	-
	**Vha13 Vacuolar H+ ATPase 13kD subunit** (CG6213)	ATP6V1G1 ATP6V1G2 ATP6V1G3 ATP6V1G2-DDX39B	-	-
	**Vha14-1 Vacuolar H+ ATPase 14kD subunit 1** (CG8210)	ATP6V1F	-	-
	**Vha14-2 Vacuolar H+ ATPase 14kD subunit 2** (CG1076)	ATP6V1F	-	-
	**Vha26 Vacuolar H+-ATPase 26kD subunit** (CG1088)	ATP6V1E1 ATP6V1E2	-	-
	**Vha36-1 Vacuolar H+ ATPase 36kD subunit 1** (CG8186)	ATP6V1D	-	-
	**Vha36-2 Vacuolar H+ ATPase 36kD subunit 2** (CG13167)	ATP6V1D	-	-

	**Vha36-3**<br>**Vacuolar H+ ATPase 36kD subunit 3**<br>(CG8310)	ATP6V1D	-	-
	**Vha44**<br>**Vacuolar H+ ATPase 44kD subunit**<br>(CG8048)	ATP6V1C1<br>ATP6V1C2	-	-
	**Vha55**<br>**Vacuolar H+-ATPase 55kD subunit**<br>(CG17369)	ATP6V1B2<br>ATP6V1B1<br>RHO	-	-
	**VhaSFD**<br>**Vacuolar H+-ATPase SFD subunit**<br>(CG17332)	ATP6V1H	-	-
**RagA-B**<br>**Ras-related GTP binding A/B**<br>(CG11968)		RRAGB<br>RRAGA<br>IL4	-	-
**RagC-D**<br>**Ras-related GTP binding C/D**<br>(CG8707)		RRAGC<br>RRAGD	-	-
**Lkb1**<br>**Lkb1 kinase**<br>(CG9374)		STK11	Ubiquitous overexpression of *Lkb1* in females leads to 6.5% increase of mean lifespan relative to control. No significant increase in lifespan was observed in male flies.	[5]
**AMPK complex**	**Alc**<br>**Alicorn**<br>(CG8057)	PRKAB1<br>PRKAB2	-	-
	**AMPKα**<br>**AMP-activated protein kinase α subunit**<br>(CG3051)	PRKAA2<br>PRKAA1	*AMPKα*-RNAi leads in approximately 20% decrease in lifespan compared to wild-type flies.	[6]
	**SNF4Aγ**<br>**SNF4/AMP-activated protein kinase gamma subunit**<br>(CG17299)	PRKAG1<br>PRKAG2<br>PRKAG3	-	-
**WDB-PP2A complex**	**Mts**<br>**Microtubule star**<br>(CG7109)	PPP2CA<br>PPP2CB	-	-
	**Wdb**<br>**Widerborst**	PPP2R5E<br>PPP2R5A	Ubiquitous overexpression of *wdb* leads to 17% and 13% increase in mean lifespan of males and	[5]

	(CG5643)	PPP2R5B	females, respectively.	
	**Pp2A-29B Protein phosphatase 2A at 29B** (CG17291)	PPP2R1A<br>PPP2R1B<br>PPP4R4<br>PPP4R1<br>RELCH	-	-
**Chrb Charybde** (CG7533)		DDIT4L<br>DDIT4	-	-
**Scyl Scylla** (CG7590)		DDIT4<br>DDIT4L	-	-
**Gig Gigas** **(**CG6975**)**		TSC2	Overexpression of *gig* under ubiquitously expressed driver extended mean lifespan at 29°C by 12%. Overexpression of *gig* in the eye or in the nervous system did not extend lifespan. Overexpression of *gig* by using the drivers that are predominantly expressed in the muscle and fat, resulted in mean lifespan extensions of 27% and 37%, respectively, at 29°C. The fat-specific drivers, when used to overexpress *gig*, also led to a mean lifespan extension of 22% and 31%, respectively, at 29°C.	[7]
**Tsc1** **(**CG6147**)**		TSC1	Overexpression of *Tsc1* under ubiquitously expressed driver extended mean lifespan at 29°C by 14%.	[7]
**Rheb Ras homolog enriched in brain** (CG1081**)**		RHEB<br>RHEBL1	Overexpression of *Rheb* in enteroblasts shortened mean lifespan by 15%. Overexpression of *Rheb* in midgut stem and progenitor cells shortened the lifespan and mean lifespan by 33%.	[8]
**Fkbp12 FK506-binding protein 12kD** (CG11001)		FKBP1A<br>FKBP1B<br>FKBP1C	-	-
**Tor Target of rapamycin** (CG5092)		MTOR<br>PRKDC	Ubiquitous overexpression of dominant-negative form of *Tor* leads to 24% and 26% increase in mean lifespan increase at 29°C and at 25°C, respectively. Overexpression of the dominant-negative form of *Tor* by using the fat-specific driver leads to 30% increase in mean lifespan at 29°C.<br><br>The median lifespan of the heteroallelic Tor^{2L7}/Tor^{k17004} mutants was increased by 20% under normal feeding conditions. Complete loss of *Tor* function results in early larval lethality.	[7]<br><br>[9]
**Lst8** (CG3004)		MLST8	-	-
**Raptor** (CG4320)		RPTOR	Muscle-specific knockdown of *raptor* leads to late pupal lethality in *Drosophila* due to an inability to eclose from the pupal case. And a large proportion of the flies that successfully eclosed died shortly afterwards. However, among the flies that survived, they generally had a normal lifespan.	[10]
**Rictor rapamycin-insensitive companion of Tor** (CG8002)		RICTOR	Cardiac-specific overexpression of *rictor* leads to 12% extension of mean lifespan.	[11]

**Sin1**<br>**SAPK-interacting protein 1**<br>(CG10105)		MAPKAP1	-	-
**Mitf**<br>(CG43369)		MITF<br>TFEB<br>TFE3<br>TFEC	*Mitf* overexpression in the nervous system led to 10 and 11% decrease in the average and median lifespan of females, respectively.	[12]
**REPTOR**<br>**Repressed by TOR**<br>(CG13624)		CREBRF	*REPTOR* knockout leads in about 59% and 40% decrease in lifespan in male and female flies, respectively.	[13]
**REPTOR-BP**<br>**REPTOR-binding partner**<br>(CG18619)		CREBL2<br>ALOX15B	*REPTOR-BP* knockout leads in about 40% and 45% decrease in lifespan in male and female flies, respectively.	[13]
**Maf1**<br>**(**CG40196)		MAF1	Gut-specific overexpression of *Maf1* mildly extended lifespan for about 2%.	[14]
**Myc**<br>(CG10798)		MYC<br>MYCL<br>MYCN<br>MXD1<br>MXD4<br>MYCLP1	*Myc* overexpression shortened median lifespan from 38 days in controls to 22 days at 29°C and from a median of 58 days in controls to 31 days at 25°C. *Myc* haploinsufficiency extends a median lifespan from 59 to 67days.	[15]
**Nclb**<br>**No child left behind**<br>(CG6751)		PWP1	-	-
**Tif-IA**<br>(CG3278)		RRN3<br>RRN3P1<br>RRN3P2	The longevity of females harboring allele *Tif-IA*^{KG}, which abolishes almost completely the *Tif-IA* expression, was robustly observed in three independent experimental trials with an average 8% extension of the median lifespan.	[16]
**Pol I***<br>12 genes<br>(FBgg0001647)	**Polr1A**<br>**RNA polymerase I subunit A**<br>(CG10122)	POLR1A	*Polr1A*$^{SH/+}$ females have about 12% extension of the lifespan.	[16]
**Pol III***<br>17 genes<br>(FBgg0001648)	**Polr3D**<br>**RNA polymerase III subunit D**<br>(CG5147)	POLR3D	Partial reduction in *Polr3D* mRNA in the adult fly gut leads to slightly extension of lifespan for approximately 5%.	[14]

References

1. Lu, J.; Temp, U.; Müller-Hartmann, A.; Esser, J.; Grönke, S.; Partridge, L. Sestrin is a key regulator of stem cell function and lifespan in response to dietary amino acids. *Nat Aging* **2021**, *1*, 60-72, doi:10.1038/s43587-020-00001-7.
2. Xi, J.; Cai, J.; Cheng, Y.; Fu, Y.; Wei, W.; Zhang, Z.; Zhuang, Z.; Hao, Y.; Lilly, M.A.; Wei, Y. The TORC1 inhibitor Nprl2 protects age-related digestive function in Drosophila. *Aging (Albany NY)* **2019**, *11*, 9811-9828, doi:10.18632/aging.102428.
3. Dulac, A.; Issa, A.R.; Sun, J.; Matassi, G.; Jonas, C.; Chérif-Zahar, B.; Cattaert, D.; Birman, S. A Novel Neuron-Specific Regulator of the V-ATPase in Drosophila. *eNeuro* **2021**, *8*, doi:10.1523/ENEURO.0193-21.2021.

4. Tian, Y.; Yu, G.; Li, K.; Du, Y.; Yuan, Z.; Gao, Y.; Fan, X.; Yang, D.; Mao, X.; Yang, M. VhaAC39-1 regulates gut homeostasis and affects the health span in Drosophila. *Mech Ageing Dev* **2022**, *204*, 111673, doi:10.1016/j.mad.2022.111673.
5. Funakoshi, M.; Tsuda, M.; Muramatsu, K.; Hatsuda, H.; Morishita, S.; Aigaki, T. A gain-of-function screen identifies wdb and lkb1 as lifespan-extending genes in Drosophila. *Biochem Biophys Res Commun* **2011**, *405*, 667-672, doi:10.1016/j.bbrc.2011.01.090.
6. Yang, S.; Long, L.H.; Li, D.; Zhang, J.K.; Jin, S.; Wang, F.; Chen, J.G. β-Guanidinopropionic acid extends the lifespan of Drosophila melanogaster via an AMP-activated protein kinase-dependent increase in autophagy. *Aging Cell* **2015**, *14*, 1024-1033, doi:10.1111/acel.12371.
7. Kapahi, P.; Zid, B.M.; Harper, T.; Koslover, D.; Sapin, V.; Benzer, S. Regulation of lifespan in Drosophila by modulation of genes in the TOR signaling pathway. *Curr Biol* **2004**, *14*, 885-890, doi:10.1016/j.cub.2004.03.059.
8. Strilbytska, O.M.; Semaniuk, U.V.; Storey, K.B.; Edgar, B.A.; Lushchak, O.V. Activation of the Tor/Myc signaling axis in intestinal stem and progenitor cells affects longevity, stress resistance and metabolism in Drosophila. *Comp Biochem Physiol B Biochem Mol Biol* **2017**, *203*, 92-99, doi:10.1016/j.cbpb.2016.09.008.
9. Luong, N.; Davies, C.R.; Wessells, R.J.; Graham, S.M.; King, M.T.; Veech, R.; Bodmer, R.; Oldham, S.M. Activated FOXO-mediated insulin resistance is blocked by reduction of TOR activity. *Cell Metab* **2006**, *4*, 133-142, doi:10.1016/j.cmet.2006.05.013.
10. Hatfield, I.; Harvey, I.; Yates, E.R.; Redd, J.R.; Reiter, L.T.; Bridges, D. The role of TORC1 in muscle development in Drosophila. *Sci Rep* **2015**, *5*, 9676, doi:10.1038/srep09676.
11. Chang, K.; Kang, P.; Liu, Y.; Huang, K.; Miao, T.; Sagona, A.P.; Nezis, I.P.; Bodmer, R.; Ocorr, K.; Bai, H. TGFB-INHB/activin signaling regulates age-dependent autophagy and cardiac health through inhibition of MTORC2. *Autophagy* **2020**, *16*, 1807-1822, doi:10.1080/15548627.2019.1704117.
12. Proshkina, E.N.; Shaposhnikov, M.V.; Shchegoleva, E.V.; Chernyshova, D.O.; Moskalev, A.A. Influence of Mitf gene overexpression on the life span of Drosophila melanogaster. *Izvestiya Komi* **2020**, *43*, 41-46, doi:DOI10.19110/1994-5655-2020-3-41-46.
13. Tiebe, M.; Lutz, M.; De La Garza, A.; Buechling, T.; Boutros, M.; Teleman, A.A. REPTOR and REPTOR-BP Regulate Organismal Metabolism and Transcription Downstream of TORC1. *Dev Cell* **2015**, *33*, 272-284, doi:10.1016/j.devcel.2015.03.013.
14. Filer, D.; Thompson, M.A.; Takhaveev, V.; Dobson, A.J.; Kotronaki, I.; Green, J.W.M.; Heinemann, M.; Tullet, J.M.A.; Alic, N. RNA polymerase III limits longevity downstream of TORC1. *Nature* **2017**, *552*, 263-267, doi:10.1038/nature25007.
15. Greer, C.; Lee, M.; Westerhof, M.; Milholland, B.; Spokony, R.; Vijg, J.; Secombe, J. Myc-dependent genome instability and lifespan in Drosophila. *PLoS One* **2013**, *8*, e74641, doi:10.1371/journal.pone.0074641.
16. Martínez Corrales, G.; Filer, D.; Wenz, K.C.; Rogan, A.; Phillips, G.; Li, M.; Feseha, Y.; Broughton, S.J.; Alic, N. Partial Inhibition of RNA Polymerase I Promotes Animal Health and Longevity. *Cell Rep* **2020**, *30*, 1661-1669.e1664, doi:10.1016/j.celrep.2020.01.017.
