# Supplementary material for: Drosophila as a Model Organism to Study Basic Mechanisms of Longevity
Source: Int J Mol Sci. 2022 Sep 24;23(19):11244. doi: 10.3390/ijms231911244 (PMC9569508; doi:10.3390/ijms231911244)
Supplement: Supplementary file 1 [file ijms-23-11244-s001.zip › Supplementary Table S3.pdf]

**Supplementary Table S3.** Summary of input signals and chromatin effector proteins for NF-kB signaling pathway controlling *Drosophila* lifespan. *Drosophila* and human protein symbols are provided according to the FlyBase annotation (March 29, 2022; http://flybase.org/). Alternative protein symbols (synonyms) are indicated within brackets.

| Components | Human orthologs | Effect(s) on lifespan | Reference(s) |
|---|---|---|---|
| **Spz**<br>**Spatzle**<br>(CG6134) | - | Downregulation of *spz* does not influence lifespan.<br><br>Heterozygous loss-of-function *spz* mutants extended median longevity by 25 and 30%, respectively. | [1]<br><br>[2] |
| **Tl**<br>**Toll**<br>(CG5490) | TLR1<br>TLR2<br>TLR3<br>TLR4<br>TLR5<br>TLR6<br>TLR7<br>TLR9<br>TLR10<br>IL1RAPL2 | Downregulation of *Tl* extended lifespan for about 12% and 2% in female and male flies, respectively. | [1] |
| **Pli**<br>**Pellino**<br>(CG5212) | PELI1<br>PELI2<br>PELI3 | - | - |
| **Myd88**<br>(CG2078) | MYD88<br>SIGIRR<br>TIRAP | - | - |
| **Tub**<br>**Tube**<br>(CG10520) | HASPIN<br>IRAK4 | - | - |
| **Pll**<br>**Pelle**<br>(CG5974) | IRAK4<br>IRAK1<br>IRAK2<br>IRAK3<br>ANKK1<br>MIR718<br>MIR6502<br>PKDCC<br>RIPK4 | - | - |
| **Cact**<br>**Cactus**<br>(CG5848) | NFKBIA<br>NFKBIB<br>NFKBIE<br>ANKRD22<br>BCL3 | Silencing the antagonist *cact* leads to about 45% reduction of lifespan in females and males.<br><br>Knockdown of *cact* pan-neuronally resulted in a dramatic reduction in the median lifespan of male and female flies for about 30% and 20%, respectively. Knockdown of *cact* in insulin-specific cells reduced median lifespan of male and female flies for about 30% and 15%, respectively. Neuroblast-specific *cact* overexpression influenced the median lifespan of male and female flies for about 16% and 4%, | [1]<br><br>[3] |

| | | | |
|---|---|---|---|
| | | respectively, suggesting a possible sex-specific effect on lifespan. | |
| **Dif**<br>**Dorsal-related immunity factor**<br>(CG6794) | RELA<br>RELB | Pan-neuronal *Dif* overexpression leads to 25% reduction of median lifespan in male and female flies. Overexpression of *Dif* in insulin-specific cells leads about 12% and 25% reduction in median lifespan of male and female flies, respectively. Neuroblast-specific *Dif* overexpression leads to 18% reduction of median lifespan in male flies and does not influence lifespan in females. | [3] |
| **Dl**<br>**Dorsal**<br>(CG6667) | RELA<br>REL | - | - |
| **PGRP-LC**<br>**Peptidoglycan recognition protein LC**<br>(CG4432) | PGLYRP1<br>PGLYRP3<br>PGLYRP4 | Overexpression of *PGRP-LC* in muscles leads to 40% and 36% reduction of the average lifespan in males and females, respectively. Overexpression of *PGRP-LC* in the fat body leads to about 90% and 84% reduction of the average lifespan in males and females, respectively. | [4] |
| **PGRP-LE**<br>**Peptidoglycan recognition protein LE**<br>(CG8995) | PGLYRP1<br>PGLYRP3<br>PGLYRP4<br>ADIPOQ | Overexpression of *PGRP-LE* in the abdominal fat body leads to about 22% and 3% reduction of the lifespan in males and females, respectively. | [5] |
| **Pirk**<br>**Poor Imd response upon knock-in**<br>(CG15678) | - | *pirk* mutant flies showed a 9.1% reduction in maximum lifespan compared with wild-type controls. | [6] |
| **Imd**<br>**Immune deficiency**<br>(CG5576) | - | Downregulation of *imd* leads to about 15% and 12% increase of mean lifespan in heterozygous males and females, respectively, and to 23% and 22% increase of mean lifespan in homozygous males and females, respectively. | [7] |
| **Diap2**<br>**Death-associated inhibitor of apoptosis 2**<br>(CG8293) | BIRC2<br>BIRC3<br>XIAP<br>BIRC7<br>BIRC8<br>NAIP<br>F11<br>F12<br>NLRC4 | - | - |
| **Eff**<br>**Effete**<br>(CG7425) | UBE2D2<br>UBE2D3<br>UBE2D1<br>UBE2D4<br>UBE2DNL<br>UBE2J1<br>UBE2T<br>UBE2U | - | - |
| **Ben**<br>**Bendless**<br>(CG18319) | UBE2N<br>UBE2NL<br>UBE2J1<br>UBE2T<br>UBE2U | *ben* mutant males show reduction of lifespan for about 22% as compared with wild-type males. This reduction could be rescued by neural expression of *hep*. | [8] |

| | | | |
|---|---|---|---|
| **Uev1A**<br>**Ubiquitin-conjugating enzyme variant 1A**<br>(CG10640) | UBE2V2<br>TMEM189-UBE2V1<br>UBE2V1 | - | - |
| **Dredd**<br>**Death related ced-3/Nedd2-like caspase**<br>(CG7486) | CASP10<br>CASP8<br>CASP9<br>CFLAR<br>CASP1<br>CARD8<br>PEA15 | Hypomorphic *Dredd* mutation leads to 12% increase of the lifespan compared to controls. *Dredd*-null flies had a 8% reduction in lifespan compared with their genetic backgrounds.<br><br>RNAi of *Dredd* had sexually antagonistic effects on lifespan, leading to 10 days increase and 15 days decrease of lifespan compared to control in male and female flies, respectively. | [6]<br><br>[9] |
| **Fadd**<br>**Fas-associated death domain**<br>(CG12297) | FADD | - | - |
| **Trbd**<br>**Trabid**<br>(CG9448) | ZRANB1<br>OTUD7A<br>OTUD7B<br>TNFAIP3<br>VCPIP1 | *trbd* mutant flies showed 37.1% reduction in maximum lifespan compared with wild-type controls.<br><br>50% of flies heterozygous for both *pirk* and *trbd* survived the 30-day mark (LT 50 = 32). A similar effect was observed in flies deficient for *pirk* in a *trbd* heterozygous background (LT 50 = 27). Just deleting *trbd* (in a *pirk* heterozygous background) had serious consequences, as 50% of flies were dead by 18 days (LT 50 = 18). More significantly however, the double mutant *pirk*; *trbd* had a dramatic reduction in life span compared to either single mutant (+/*pirk*; *trbd* or *pirk*; +/*trbd*) or double heterozygote (+/*pirk*; +/*trbd*) since 50% of flies were dead before the 14-day mark (LT 50 = 14). This phenomenon was suppressed in *dredd*; *pirk*; *trbd* flies (LT 50 = 37) where IMD was inactive. | [6]<br><br>[10] |
| **Tak1**<br>**TGF-β activated kinase 1**<br>(CG18492) | MAP3K7<br>RIPK1<br>RIPK3<br>ANKK1<br>DSTYK<br>FPGT-TNNI3K<br>RIPK4<br>TNNI3K | Knock-down of *Tak1* in intestinal cells causes about 5% extension of mean longevity compared with control. | [11] |
| **Tab2**<br>**TAK1-associated binding protein 2**<br>(CG7417) | TAB2<br>TAB3<br>TNFRSF1A | - | - |
| **Key**<br>**Kenny**<br>(CG16910) | IKBKG<br>OPTN<br>CEP55<br>TNIP2 | - | - |
| **IKKβ**<br>**I-kappaB kinase β**<br>(CG4201) | CHUK<br>IKBKB<br>IKBKE<br>ERLIN1 | - | - |

| | | | |
|---|---|---|---|
| **Casp**<br>**Caspar**<br>(CG8400) | FAF1 | *casp* mutants have shorter lifespans than the controls, with the change in median lifespan being shorter by 19%. | [12] |
| **Rel**<br>**Relish**<br>(CG11992) | NFKB1<br>NFKB2 | Pan-neuronal *Rel* overexpression leads in 25% reduction of median lifespan in male and female flies. *Rel* overexpression in insulin-producing cells and neuroblasts leads about 1% and 11% reduction of the lifespan of male and female flies, respectively. Pan-neuronal overexpression of the constitutively-active Rel domain of Rel reduced the median lifespan for about 25% in males and females. Overexpression of the constitutively-active Rel domain of Rel in insulin-producing cells leads reduction of median lifespan for about 20% in male and female flies. Overexpression of the constitutively-active Rel domain of Rel in neuroblasts leads reduction of median lifespan for about 15% and 9% in male and female flies, respectively.<br><br>*Rel*-null flies had a statistically significant reduction (for about 23%) in lifespan compared with their genetic backgrounds. RNAi-dependent silencing of *Rel* in glia, neurons and intestine resulted in about 60%, 38% and 35% increase of lifespan, respectively.<br><br>Ubiquitous overexpression of *Rel* shortened lifespan for about 30% and 39% in female and male flies, respectively. Overexpression of *Rel* in fat body shortened lifespan for about 24% and 18% in female and male flies, respectively. Underexpression of *Rel* shortened lifespan of females for about 4% and extended lifespan of males for about 3%. Underexpression of *Rel* in fat body extended lifespan for about 11% and 13% in female and male flies, respectively | [3]<br><br>[6]<br><br>[13] |
| **Tg**<br>**Transglutaminase**<br>(CG7356) | TGM1<br>TGM4<br>F13A1<br>TGM2<br>TGM5<br>EPB42<br>TGM3<br>TGM6<br>TGM7<br>CCNI | *Tg* mutant flies showed a 24.2% reduction in maximum lifespan compared with wild-type controls. | [6] |
| **Charon**<br>(CG5118) | ANKRD7<br>ANKRD26P1<br>ASB7<br>ASB15<br>ASB18<br>BCL3<br>NFKBIE<br>POTEA<br>POTEB2<br>POT<br>EB3 | Long-term GeneSwitch-mediated depletion of pickle in enteroblasts and enterocytes caused a significant reduction in median lifespan (6-10%). | [14] |

| | | | |
|---|---|---|---|
| | POTEB<br>POTEC<br>POTED<br>POTEE<br>POTEF<br>POTEG<br>POTEH<br>POTEI<br>POTEJ<br>POTEM | | |
| **Zfh1**<br>**Zn finger homeodomain 1**<br>(CG1322) | ZEB2<br>ZEB1 | - | - |
| **Nub**<br>**Nubbin**<br>(CG34395) | POU2F1<br>POU2F2<br>POU5F1B<br>POU5F1<br>POU5F2<br>CCHCR1 | Importantly, *nub* mutant flies exhibited a considerably reduced lifespan ($T_{50}$ = 21.7 days) compared to OregonR ($T_{50}$ = 68.5 days). | [15] |
| **Cad**<br>**Caudal**<br>(CG1759) | CDX2<br>CDX1<br>CDX4<br>URAD | There was no significant increase (but a minor reduction) in the longevity of flies with *cad* depletion compared to the control flies. | [16] |
| **Polybromo**<br>(CG11375) | PBRM1 | - | - |
| **Akirin**<br>(CG8580) | AKIRIN2<br>AKIRIN1 | - | - |
| **Gcn5**<br>**Gcn5 acetyltransferase**<br>(CG4107) | KAT2A<br>KAT2B | - | - |

## References


1. Fabian, D.K.; Garschall, K.; Klepsatel, P.; Santos-Matos, G.; Sucena, É.; Kapun, M.; Lemaitre, B.; Schlötterer, C.; Arking, R.; Flatt, T. Evolution of longevity improves immunity in Drosophila. *Evol Lett* **2018**, *2*, 567-579, doi:10.1002/evl3.89.
2. Zhan, L.; Xie, Q.; Tibbetts, R.S. Opposing roles of p38 and JNK in a Drosophila model of TDP-43 proteinopathy reveal oxidative stress and innate immunity as pathogenic components of neurodegeneration. *Hum Mol Genet* **2015**, *24*, 757-772, doi:10.1093/hmg/ddu493.
3. Khor, S.; Cai, D. Control of lifespan and survival by Drosophila NF-κB signaling through neuroendocrine cells and neuroblasts. *Aging (Albany NY)* **2020**, *12*, 24604-24622, doi:10.18632/aging.104196.
4. DeVeale, B.; Brummel, T.; Seroude, L. Immunity and aging: the enemy within? *Aging Cell* **2004**, *3*, 195-208, doi:10.1111/j.1474-9728.2004.00106.x.
5. Libert, S.; Chao, Y.; Chu, X.; Pletcher, S.D. Trade-offs between longevity and pathogen resistance in Drosophila melanogaster are mediated by NFkappaB signaling. *Aging Cell* **2006**, *5*, 533-543, doi:10.1111/j.1474-9726.2006.00251.x.
6. Kounatidis, I.; Chtarbanova, S.; Cao, Y.; Hayne, M.; Jayanth, D.; Ganetzky, B.; Ligoxygakis, P. NF-κB Immunity in the Brain Determines Fly Lifespan in Healthy Aging and Age-Related Neurodegeneration. *Cell Rep* **2017**, *19*, 836-848, doi:10.1016/j.celrep.2017.04.007.

7. Lin, Y.R.; Parikh, H.; Park, Y. Stress resistance and lifespan enhanced by downregulation of antimicrobial peptide genes in the Imd pathway. *Aging (Albany NY)* **2018**, *10*, 622-631, doi:10.18632/aging.101417.
8. Ma, X.; Li, W.; Yu, H.; Yang, Y.; Li, M.; Xue, L.; Xu, T. Bendless modulates JNK-mediated cell death and migration in Drosophila. *Cell Death Differ* **2014**, *21*, 407-415, doi:10.1038/cdd.2013.154.
9. Parker, G.A.; Kohn, N.; Spirina, A.; McMillen, A.; Huang, W.; Mackay, T.F.C. Genetic Basis of Increased Lifespan and Postponed Senescence in Drosophila melanogaster. *G3 (Bethesda)* **2020**, *10*, 1087-1098, doi:10.1534/g3.120.401041.
10. Fernando, M.D.; Kounatidis, I.; Ligoxygakis, P. Loss of Trabid, a new negative regulator of the drosophila immune-deficiency pathway at the level of TAK1, reduces life span. *PLoS Genet* **2014**, *10*, e1004117, doi:10.1371/journal.pgen.1004117.
11. Ji, S.; Luo, Y.; Cai, Q.; Cao, Z.; Zhao, Y.; Mei, J.; Li, C.; Xia, P.; Xie, Z.; Xia, Z.; et al. LC Domain-Mediated Coalescence Is Essential for Otu Enzymatic Activity to Extend Drosophila Lifespan. *Mol Cell* **2019**, *74*, 363-377.e365, doi:10.1016/j.molcel.2019.02.004.
12. Kaduskar, B.; Trivedi, D.; Ratnaparkhi, G.S. Caspar SUMOylation regulates Drosophila lifespan. *MicroPubl Biol* **2020**, *2020*, doi:10.17912/micropub.biology.000288.
13. Badinloo, M.; Nguyen, E.; Suh, W.; Alzahrani, F.; Castellanos, J.; Klichko, V.I.; Orr, W.C.; Radyuk, S.N. Overexpression of antimicrobial peptides contributes to aging through cytotoxic effects in Drosophila tissues. *Arch Insect Biochem Physiol* **2018**, *98*, e21464, doi:10.1002/arch.21464.
14. Morris, O.; Liu, X.; Domingues, C.; Runchel, C.; Chai, A.; Basith, S.; Tenev, T.; Chen, H.; Choi, S.; Pennetta, G.; et al. Signal Integration by the IκB Protein Pickle Shapes Drosophila Innate Host Defense. *Cell Host Microbe* **2016**, *20*, 283-295, doi:10.1016/j.chom.2016.08.003.
15. Dantoft, W.; Lundin, D.; Esfahani, S.S.; Engström, Y. The POU/Oct Transcription Factor Pdm1/nub Is Necessary for a Beneficial Gut Microbiota and Normal Lifespan of Drosophila. *J Innate Immun* **2016**, *8*, 412-426, doi:10.1159/000446368.
16. Wu, K.; Tang, Y.; Zhang, Q.; Zhuo, Z.; Sheng, X.; Huang, J.; Ye, J.; Li, X.; Liu, Z.; Chen, H. Aging-related upregulation of the homeobox gene caudal represses intestinal stem cell differentiation in Drosophila. *PLoS Genet* **2021**, *17*, e1009649, doi:10.1371/journal.pgen.1009649.
