# Supplementary material for: Drosophila as a Model Organism to Study Basic Mechanisms of Longevity
Source: Int J Mol Sci. 2022 Sep 24;23(19):11244. doi: 10.3390/ijms231911244 (PMC9569508; doi:10.3390/ijms231911244)
Supplement: Supplementary file 1 [file ijms-23-11244-s001.zip › Supplementary Table S4.pdf]

Supplementary Table 4. Summary of input signals and chromatin effector proteins for JNK signaling pathway controlling *Drosophila* lifespan. *Drosophila* and human protein symbols are provided according to the FlyBase annotation (March 29, 2022; http://flybase.org/). Alternative protein symbols (synonyms) are indicated within brackets.

**Components**	**Human orthologs**	**Effect(s) on lifespan**	**Reference(s)**
**Egr**<br>**Eiger**<br>(CG12919)	EDA<br>TNFSF12-<br>TNFSF13<br>TNFSF13B<br>TNFSF13<br>TNFSF12	*egr* mutant flies displayed of about 40% increase in lifespan compared to wild-type flies.	[1]
**Wgn**<br>**Wengen**<br>(CG6531)	NGFR<br>TNFRSF4	-	-
**Grnd**<br>**Grindelwald**<br>(CG10176)	-	-	-
**CYLD**<br>**Cylindromatosis**<br>(CG5603)	CYLD	*CYLD* mutant flies show about 42% reduction of lifespan as compared with wild-type or heterozygous *CYLD* flies. This reduction was largely rescued by one copy of *CYLDRes* and fully rescued by ubiquitous expression of *Bsk*.	[2]
**Traf6**<br>**TNF-receptor-associated factor 6**<br>(CG10961)	TRAF6<br>TRAF5<br>TRAF7	Knock-down of *Traf6* in intestinal cells causes about 24% reduction of mean longevity compared with control.	[3]
**Traf4**<br>**TNF-receptor-associated factor 4**<br>(CG3048)	TRAF4<br>TRAF1<br>TRAF2<br>TRAF3<br>TRAF5<br>TRAF7	-	-
**Msn**<br>**Misshapen**<br>(CG16973)	TNIK<br>MINK1<br>MAP4K4<br>NRK	RNAi of *msn* decreased lifespan in about 11days in female flies compared to control.<br><br>Overexpression of *msn* in eye and antennal imaginal disks causes about 70% reduction of lifespan compared with control.	[4]<br><br>[5]
**Tak1**<br>**TGF-β activated kinase 1**<br>(CG18492)	MAP3K7<br>RIPK1<br>RIPK3<br>ANKK1<br>DSTYK<br>FPGT-<br>TNNI3K<br>RIPK4<br>TNNI3K	Knock-down of *Tak1* in intestinal cells causes about 5% extension of mean longevity compared with control.	[3]
**Tab2**<br>**TAK1-associated binding protein 2**<br>(CG7417)	TAB2<br>TAB3<br>TNFRSF1A	-	-
**Slpr**<br>**Slipper**<br>(CG2272)	MAP3K9<br>MAP3K10<br>MAP3K11<br>MAP3K21<br>RIPK1	Misexpression of *slpr* in muscles and salivary glands or in hemocytes causes 79% or 71% reduction in mean longevity, respectively, compared with control.	[6]

		RIPK2<br>ANKK1<br>DSTYK<br>FPGT-<br>TNNI3K<br>RIPK4<br>TNNI3K		
**Ask1**<br>**Apoptotic signal-regulating kinase 1**<br>(CG4720)		MAP3K15<br>MAP3K5<br>MAP3K3	-	-
**Mekk1**<br>(CG7717)		MAP3K4<br>MAP3K2<br>MAP3K3<br>MAP3K19	-	
**Wnd**<br>**Wallenda**<br>(CG8789)		MAP3K13<br>MAP3K12	-	-
**Mkk4**<br>**MAP kinase kinase 4**<br>(CG9738)		MAP2K4<br>LOC100996<br>792	-	-
**Hep**<br>**Hemipterous**<br>(CG4353)		MAP2K7<br>LOC100996<br>792	Neuronal overexpression of *hep* extended lifespan of about 25% compared to control.<br><br>Activation of *hep* expression in intestinal stem cells and enteroblasts results in about 6% reduction of mean lifespan in male and female flies compared with control.<br><br>Overexpression of *hep* in intestinal stem cells and enteroblasts causes 51% and 25% reduction of mean lifespan in male and female flies, respectively, compared to control.	[7]<br><br>[8]<br><br>[9]
**STRIATIN-INTERACTING PHOSPHATASE AND KINASE COMPLEX STRIPAK**<br>(FBgg0000915)	**Ccm3**<br>**Cerebral cavernous malformation 3**<br>(CG5073)	PDCD10	-	-
	**Cka**<br>**Connector of kinase to AP-1**<br>(CG7392)	STRN3<br>STRN<br>STRN4	-	-
	**Fgop2**<br>**Fibroblast growth factor receptor 1 oncogene partner 2**<br>(CG10158)	FGFR1OP2<br>SIKE1	-	-
	**Mob4**<br>**MOB kinase activator 4**<br>(CG3403)	MOB4<br>HSPE1-<br>MOB4	-	-
	**Mts**<br>**Microtubule star**	PPP2CA<br>PPP2CB<br>PPP5D1	-	-

	(CG7109)			
	**Naus**<br>**Nausicaa**<br>(CG10915)	CTTNBP2NL<br>CTTNBP2<br>FILIP1L<br>FILIP1<br>LUZP1	-	-
	**Pp2A-29B**<br>**Protein phosphatase 2A at 29B**<br>(CG17291)	PPP2R1A<br>PPP2R1B<br>PPP4R4<br>PPP4R1<br>RELCH	-	-
	**Slmap**<br>**Sarcolemma associated protein**<br>(CG17494)	SLMAP<br>CCDC136<br>TRAF3IP3<br>RNF8	-	-
	**Strip**<br>**Striatin interacting protein**<br>(CG11526)	STRIP1<br>STRIP2	-	-
**Src42A**<br>**Src oncogene at 42A**<br>(CG44128)		FRK<br>PTK6<br>SRMS	-	-
**Src64B**<br>**Src oncogene at 64B**<br>(CG7524)		FYN<br>SRC<br>YES1<br>FGR<br>BLK<br>HCK<br>LCK<br>LYN<br>PTK6<br>SRMS<br>SLA2<br>SLA	-	-
**Btk**<br>**Bruton tyrosine kinase**<br>(CG8049)		TEC<br>TXK<br>ITK<br>BTK<br>BMX<br>PTK6<br>SRMS	The *Btk* mutation reduced adult longevity to 11% of wild-type. Overexpression of Btk during the late larval and pupal stages prolonged the adult lifespan of *Btk* mutants to 53% and 76% of the wild-type level.	[10]
**Dok**<br>**Downstream of kinase**<br>(CG2079)		DOK2<br>DOK1<br>DOK3<br>DOK4<br>DOK5<br>DOK6<br>DOK7	-	-
**Shark**<br>**SH2 ankyrin repeat kinase**<br>(CG18247)		ZAP70<br>SYK	-	-
**Rac1**<br>(CG2248)		RAC1<br>RAC2<br>RAC3<br>RHOBTB3	Knockdown of *Rac1* in neurons did not affect life span.	[11]

	RHOF<br>RND1<br>RND2<br>RND3		
**Puc**<br>**Puckered**<br>(CG7850)	DUSP10<br>DUSP8<br>DUSP16<br>DUSP1<br>DUSP4<br>DUSP2<br>DUSP5<br>EPM2A<br>STYXL1	Heterozygosity of two *puc* loss-of-function alleles showed a significantly longer lifespan (of about 48% and 62%) than wild-type flies. This phenotype was reduced in puc^{E69} heterozygotes in a hep^{1} hemizygous backgound.<br><br>Long-lived phenotype in *puc* mutants can be reverted to wild-type levels in the back-ground of *foxo* loss-of-function allele heterozygotes	[7]<br><br>[12]
**Prx2**<br>**Peroxiredoxin 2**<br>(CG1633)	PRDX2<br>PRDX1	Neuronal overexpression of *Prx2* in neurons increased lifespan of about 13% compared to wild-type flies. Neuronal knockdown of *Prx2* as well as the loss-of-function mutation caused about 8% and 14% reduction in lifespan, respectively. Induced expression of *Prx2* in adult neurons extended lifespan by 26% in females and 29% in males compared with the control.<br><br>Overexpression of *Prx2* in intestinal stem cells results in 12% and 20% extension of mean lifespan in male and female flies, respectively, compared with control.	[13]<br><br>[8]
**Bsk**<br>**Basket**<br>(CG5680)	MAPK8<br>MAPK10<br>MAPK9	Strong inhibition of *bsk* expression in intestinal stem cells and enteroblasts results in 16% and 10% reduction of mean lifespan in male and female flies, respectively, compared with control. Induced inhibition of *bsk* in intestine using RNAi and dominant-negative Bsk causes 12% and 14% extension of mean lifespan, respectively, compared with uninduced flies.	[8]
**Foxo**<br>**Forkhead box, sub-group O**<br>(CG3143)	FOXO3<br>FOXO1<br>FOXO4	Heterozygous *foxo* males and females showed decrease of mean lifespan of about 5% and increase of mean lifespan of 29%, respectively.<br><br>Overexpression of *foxo* in adult head fat body increased the median lifespan of male and female flies of about 24% and 16%, respectively. Pan-neuronal overexpresion of *foxo* as well as its overexpression in fat body, neurolemma and glial cells had no significant effect on lifespan.<br><br>Overexpression of *foxo* in muscles extends the median lifespan of male flies of about 23%.<br><br>Induced expression of *foxo* in the adult fat body increased lifespan of female flies by 20 to 50%.<br><br>Heterozygosity for $foxo^{21}$ or $foxo^{25}$ does not affect life span in wild-type backgrounds.<br><br>Activation of Foxo expression in intestinal stem cells and enteroblasts results in 6% and 4% reduction of mean lifespan in males and females, respectively, compared with control.	[14]<br><br>[15]<br><br>[16]<br><br>[17]<br><br>[12]<br><br>[8]

**AP-1 transcription factor**	**Kay**<br>**Kayak**<br>(CG33956)	FOS<br>FOSL1<br>FOSL2<br>FOSB	*P*-element mutation in the *kay* gene was associated with increased lifespan.	[18]
	**Jra**<br>**Jun-related antigen**<br>(CG2275)	JUND<br>JUN<br>JUNB	Reduction of *Jra* activity in the whole animal or in the gut in the absence of infection does not affect lifespan, suggesting that Jra is haplo-sufficient in this context.	[19]
**Stat92E**<br>**Signal-transducer and activator of transcription protein at 92E**<br>(CG4257)		STAT5B<br>STAT5A<br>STAT6<br>STAT1<br>STAT2<br>STAT3<br>STAT4	Stat92E mutation exhibited shortened lifespans compared with wild-type control flies of about 20%.	[20]

References

1. Schneider, D.S.; Ayres, J.S.; Brandt, S.M.; Costa, A.; Dionne, M.S.; Gordon, M.D.; Mabery, E.M.; Moule, M.G.; Pham, L.N.; Shirasu-Hiza, M.M. Drosophila eiger mutants are sensitive to extracellular pathogens. *PLoS Pathog* **2007**, *3*, e41, doi:10.1371/journal.ppat.0030041.
2. Xue, L.; Igaki, T.; Kuranaga, E.; Kanda, H.; Miura, M.; Xu, T. Tumor suppressor CYLD regulates JNK-induced cell death in Drosophila. *Dev Cell* **2007**, *13*, 446-454, doi:10.1016/j.devcel.2007.07.012.
3. Ji, S.; Luo, Y.; Cai, Q.; Cao, Z.; Zhao, Y.; Mei, J.; Li, C.; Xia, P.; Xie, Z.; Xia, Z.; et al. LC Domain-Mediated Coalescence Is Essential for Otu Enzymatic Activity to Extend Drosophila Lifespan. *Mol Cell* **2019**, *74*, 363-377.e365, doi:10.1016/j.molcel.2019.02.004.
4. Parker, G.A.; Kohn, N.; Spirina, A.; McMillen, A.; Huang, W.; Mackay, T.F.C. Genetic Basis of Increased Lifespan and Postponed Senescence in Drosophila melanogaster. *G3 (Bethesda)* **2020**, *10*, 1087-1098, doi:10.1534/g3.120.401041.
5. Mishra, A.K.; Sachan, N.; Mutsuddi, M.; Mukherjee, A. Kinase active Misshapen regulates Notch signaling in Drosophila melanogaster. *Exp Cell Res* **2015**, *339*, 51-60, doi:10.1016/j.yexcr.2015.09.021.
6. Nakayama, M.; Ishibashi, T.; Ishikawa, H.O.; Sato, H.; Usui, T.; Okuda, T.; Yashiro, H.; Ishikawa, H.; Taikou, Y.; Minami, A.; et al. A gain-of-function screen to identify genes that reduce lifespan in the adult of Drosophila melanogaster. *BMC Genet* **2014**, *15*, 46, doi:10.1186/1471-2156-15-46.
7. Wang, M.C.; Bohmann, D.; Jasper, H. JNK signaling confers tolerance to oxidative stress and extends lifespan in Drosophila. *Dev Cell* **2003**, *5*, 811-816, doi:10.1016/s1534-5807(03)00323-x.
8. Biteau, B.; Karpac, J.; Supoyo, S.; Degennaro, M.; Lehmann, R.; Jasper, H. Lifespan extension by preserving proliferative homeostasis in Drosophila. *PLoS Genet* **2010**, *6*, e1001159, doi:10.1371/journal.pgen.1001159.
9. Biteau, B.; Hochmuth, C.E.; Jasper, H. JNK activity in somatic stem cells causes loss of tissue homeostasis in the aging Drosophila gut. *Cell Stem Cell* **2008**, *3*, 442-455, doi:10.1016/j.stem.2008.07.024.
10. Hamada, N.; Bäckesjö, C.M.; Smith, C.I.; Yamamoto, D. Functional replacement of Drosophila Btk29A with human Btk in male genital development and survival. *FEBS Lett* **2005**, *579*, 4131-4137, doi:10.1016/j.febslet.2005.06.042.

11. Kikuchi, M.; Sekiya, M.; Hara, N.; Miyashita, A.; Kuwano, R.; Ikeuchi, T.; Iijima, K.M.; Nakaya, A. Disruption of a RAC1-centred network is associated with Alzheimer's disease pathology and causes age-dependent neurodegeneration. *Hum Mol Genet* **2020**, *29*, 817-833, doi:10.1093/hmg/ddz320.
12. Wang, M.C.; Bohmann, D.; Jasper, H. JNK extends life span and limits growth by antagonizing cellular and organism-wide responses to insulin signaling. *Cell* **2005**, *121*, 115-125, doi:10.1016/j.cell.2005.02.030.
13. Lee, K.S.; Iijima-Ando, K.; Iijima, K.; Lee, W.J.; Lee, J.H.; Yu, K.; Lee, D.S. JNK/FOXO-mediated neuronal expression of fly homologue of peroxiredoxin II reduces oxidative stress and extends life span. *J Biol Chem* **2009**, *284*, 29454-29461, doi:10.1074/jbc.M109.028027.
14. Nielsen, M.D.; Luo, X.; Biteau, B.; Syverson, K.; Jasper, H. 14-3-3 Epsilon antagonizes FoxO to control growth, apoptosis and longevity in Drosophila. *Aging Cell* **2008**, *7*, 688-699, doi:10.1111/j.1474-9726.2008.00420.x.
15. Hwangbo, D.S.; Gershman, B.; Gersham, B.; Tu, M.P.; Palmer, M.; Tatar, M. Drosophila dFOXO controls lifespan and regulates insulin signalling in brain and fat body. *Nature* **2004**, *429*, 562-566, doi:10.1038/nature02549.
16. Demontis, F.; Perrimon, N. FOXO/4E-BP signaling in Drosophila muscles regulates organism-wide proteostasis during aging. *Cell* **2010**, *143*, 813-825, doi:10.1016/j.cell.2010.10.007.
17. Giannakou, M.E.; Goss, M.; Jünger, M.A.; Hafen, E.; Leevers, S.J.; Partridge, L. Long-lived Drosophila with overexpressed dFOXO in adult fat body. *Science* **2004**, *305*, 361, doi:10.1126/science.1098219.
18. Magwire, M.M.; Yamamoto, A.; Carbone, M.A.; Roshina, N.V.; Symonenko, A.V.; Pasyukova, E.G.; Morozova, T.V.; Mackay, T.F. Quantitative and molecular genetic analyses of mutations increasing Drosophila life span. *PLoS Genet* **2010**, *6*, e1001037, doi:10.1371/journal.pgen.1001037.
19. Soory, A.; Ratnaparkhi, G.S. SUMOylation of Jun fine-tunes the Drosophila gut immune response. *PLoS Pathog* **2022**, *18*, e1010356, doi:10.1371/journal.ppat.1010356.
20. Larson, K.; Yan, S.J.; Tsurumi, A.; Liu, J.; Zhou, J.; Gaur, K.; Guo, D.; Eickbush, T.H.; Li, W.X. Heterochromatin formation promotes longevity and represses ribosomal RNA synthesis. *PLoS Genet* **2012**, *8*, e1002473, doi:10.1371/journal.pgen.1002473.
