# Supplementary figures and images for: Drosophila as a Model Organism to Study Basic Mechanisms of Longevity
Source: Int J Mol Sci. 2022 Sep 24;23(19):11244. doi: 10.3390/ijms231911244 (PMC9569508; doi:10.3390/ijms231911244)

A

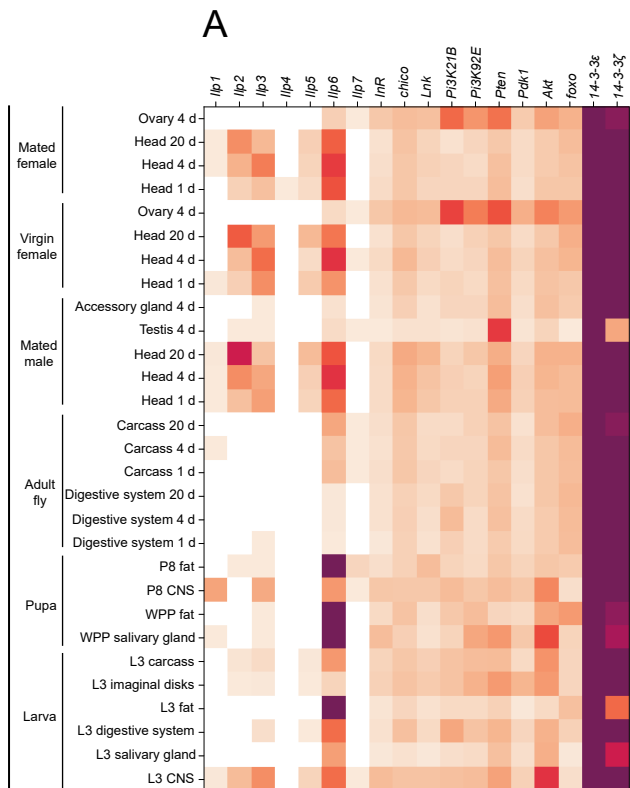

Effect on lifespan

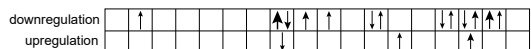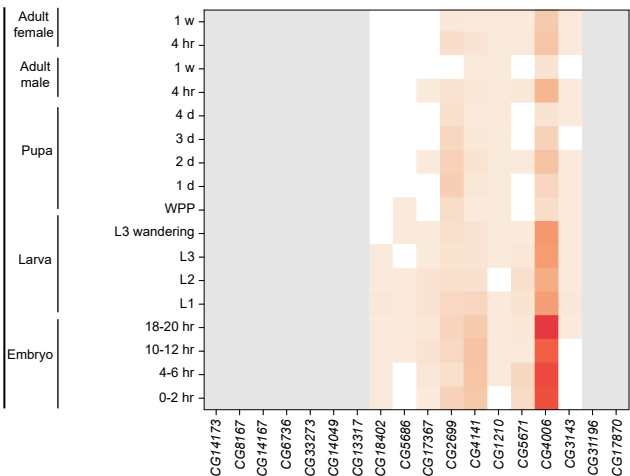

B

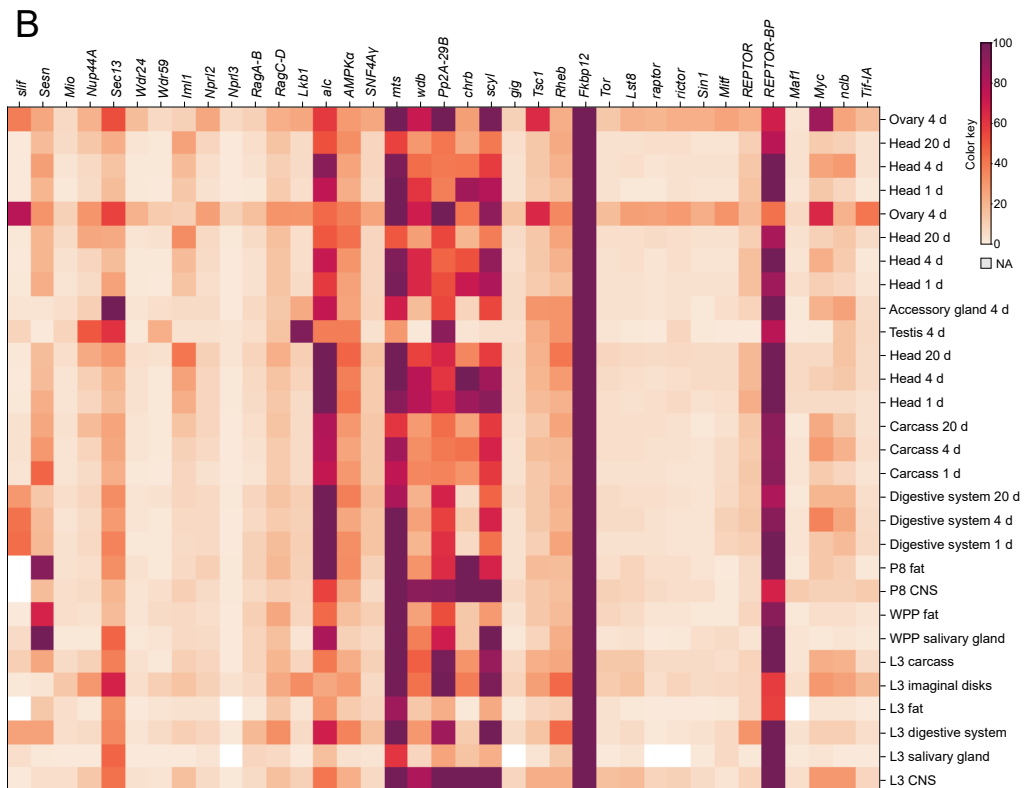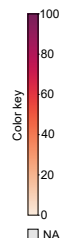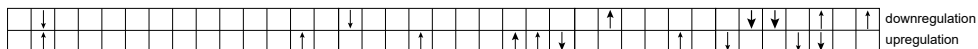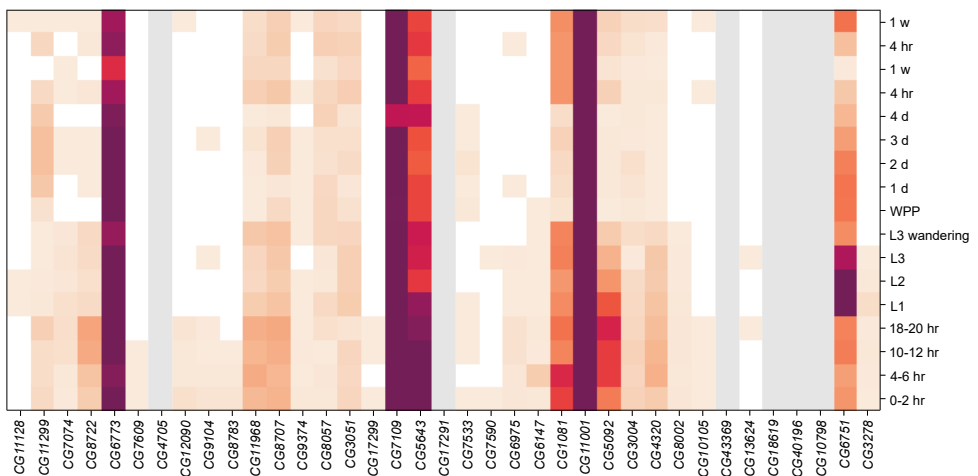

Supplement: Supplementary file 1 [file ijms-23-11244-s001.zip › Supplementary Figure S1.pdf]

**A****Anatomy**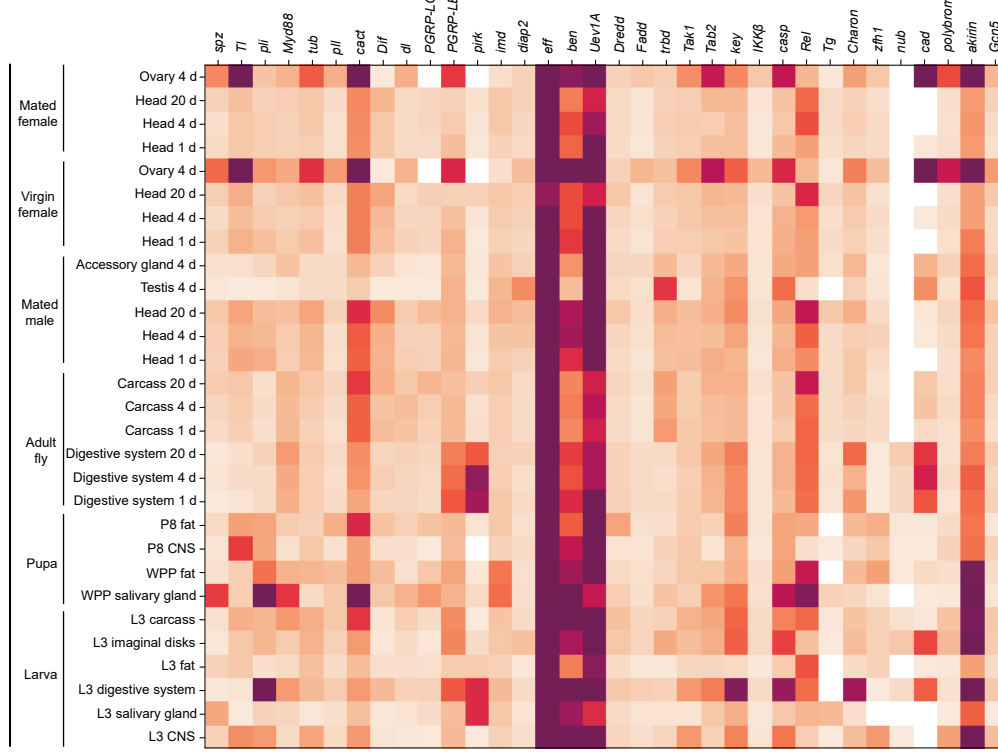**Effect on lifespan**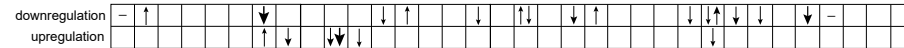**Proteome**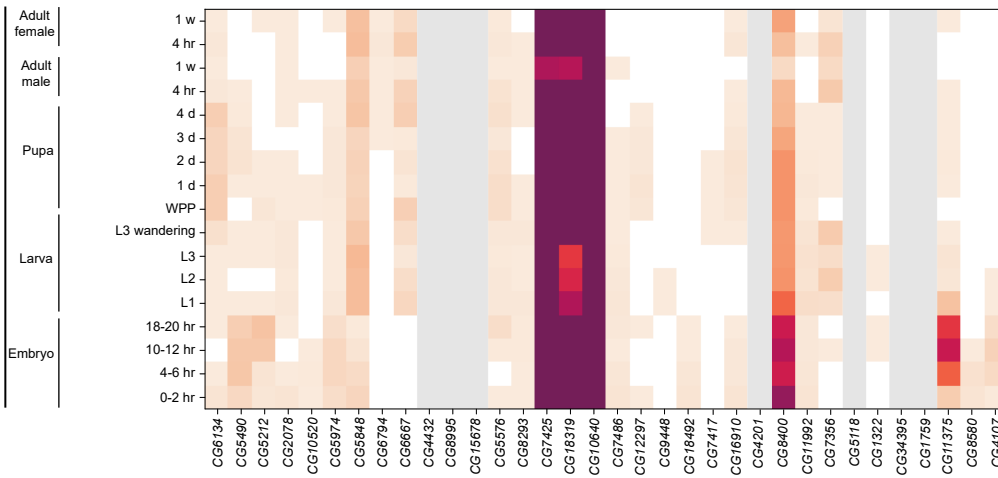**B**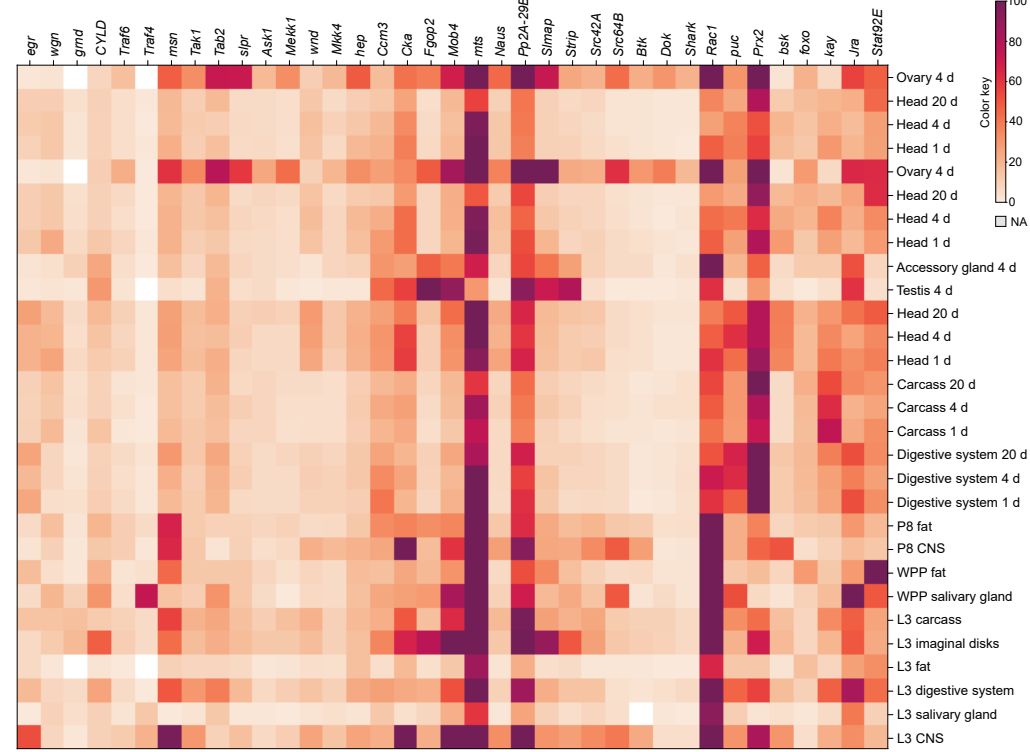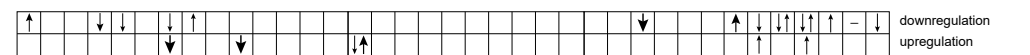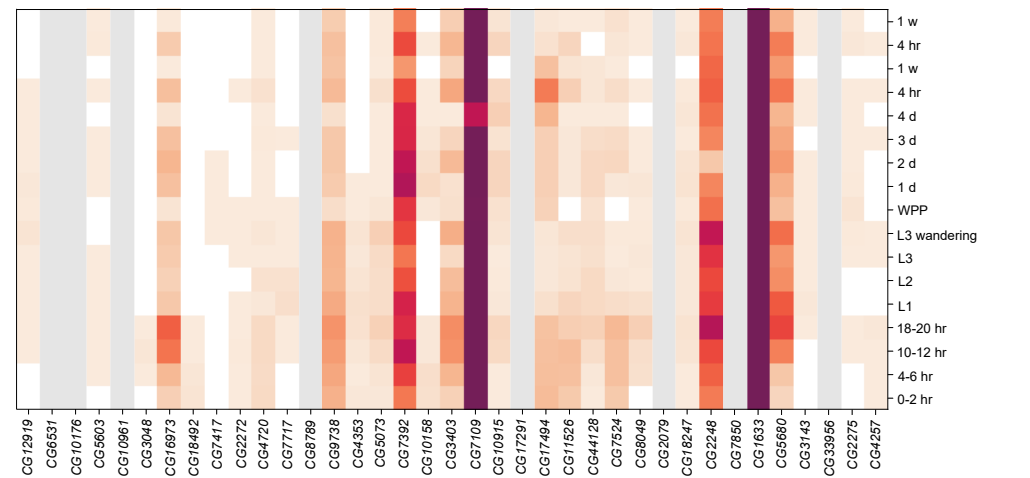

Supplement: Supplementary file 1 [file ijms-23-11244-s001.zip › Supplementary Figure S2.pdf]
